# Antimicrobial Resistance Profiling and Phylogenetic Analysis of *Neisseria gonorrhoeae* Clinical Isolates From Kenya in a Resource-Limited Setting

**DOI:** 10.3389/fmicb.2021.647565

**Published:** 2021-07-27

**Authors:** Meshack Juma, Arun Sankaradoss, Redcliff Ndombi, Patrick Mwaura, Tina Damodar, Junaid Nazir, Awadhesh Pandit, Rupsy Khurana, Moses Masika, Ruth Chirchir, John Gachie, Sudhir Krishna, Ramanathan Sowdhamini, Omu Anzala, Iyer S. Meenakshi

**Affiliations:** ^1^KAVI Institute of Clinical Research, University of Nairobi, Nairobi, Kenya; ^2^National Centre for Biological Sciences, Tata Institute of Fundamental Research (TIFR), Bengaluru, India; ^3^School of Interdisciplinary Life Sciences, Indian Institute of Technology Goa, Ponda, India

**Keywords:** nanopore sequencing, whole-genome sequencing, antimicrobial resistance, AMR prediction, sequence-typing

## Abstract

**Background:**

Africa has one of the highest incidences of gonorrhea. *Neisseria gonorrhoeae* is gaining resistance to most of the available antibiotics, compromising treatment across the world. Whole-genome sequencing (WGS) is an efficient way of predicting AMR determinants and their spread in the population. Recent advances in next-generation sequencing technologies like Oxford Nanopore Technology (ONT) have helped in the generation of longer reads of DNA in a shorter duration with lower cost. Increasing accuracy of base-calling algorithms, high throughput, error-correction strategies, and ease of using the mobile sequencer MinION in remote areas lead to its adoption for routine microbial genome sequencing. To investigate whether MinION-only sequencing is sufficient for WGS and downstream analysis in resource-limited settings, we sequenced the genomes of 14 suspected *N. gonorrhoeae* isolates from Nairobi, Kenya.

**Methods:**

Using WGS, the isolates were confirmed to be cases of *N. gonorrhoeae* (*n* = 9), and there were three co-occurrences of *N. gonorrhoeae* with *Moraxella osloensis* and *N. meningitidis* (*n* = 2). *N. meningitidis* has been implicated in sexually transmitted infections in recent years. The near-complete *N. gonorrhoeae* genomes (*n* = 10) were analyzed further for mutations/factors causing AMR using an in-house database of mutations curated from the literature.

**Results:**

We observe that ciprofloxacin resistance is associated with multiple mutations in both gyrA and parC. Mutations conferring tetracycline (*rpsJ*) and sulfonamide (*folP*) resistance and plasmids encoding beta-lactamase were seen in all the strains, and *tet(M)*-containing plasmids were identified in nine strains. Phylogenetic analysis clustered the 10 isolates into clades containing previously sequenced genomes from Kenya and countries across the world. Based on homology modeling of AMR targets, we see that the mutations in GyrA and ParC disrupt the hydrogen bonding with quinolone drugs and mutations in FolP may affect interaction with the antibiotic.

**Conclusion:**

Here, we demonstrate the utility of mobile DNA sequencing technology in producing a consensus genome for sequence typing and detection of genetic determinants of AMR. The workflow followed in the study, including AMR mutation dataset creation and the genome identification, assembly, and analysis, can be used for any clinical isolate. Further studies are required to determine the utility of real-time sequencing in outbreak investigations, diagnosis, and management of infections, especially in resource-limited settings.

## Introduction

Gonorrhea is one of the most common sexually transmitted infections (STIs), which is caused by *Neisseria gonorrhoeae* (gonococci). It is estimated to have affected 87 million people (between the ages 15 and 49) worldwide, 11.4 million in the African region, in 2016 [World Health Organisation (WHO), 2018]. Gonorrhea can cause epididymitis in men and pelvic inflammatory disease in women, which can result in infertility and ectopic pregnancies. It is also implicated in neonatal ophthalmia, which can result in blindness, and increased acquisition and transmission of other STIs (Unemo and Dillon, 2011). WHO included *N. gonorrhoeae* in its list of AMR “priority pathogens” in 2017 [World Health Organisation (WHO), 2017] and has projected a 90% reduction in global gonorrhea incidence by 2030 [World Health Organisation (WHO), 2018]. Although *N. gonorrhoeae* infections are treated with antibiotics, gonococci have acquired resistance to most of the antibiotics used to treat gonorrhea, and despite two clinical trials, no vaccines are available (Wi et al., 2017). Increasing antimicrobial resistance threatens effective treatment and control.

Many strains are developing resistance to ceftriaxone and azithromycin, which are used as the last options of the first-line therapies of gonorrhea (Unemo and Nicholas, 2012). However, with isolates showing increased MICs to both these antibiotics, this is not viable as a long-term treatment (Papp et al., 2017). Many countries in the African region have reported decreased susceptibility to drugs like ceftriaxone, azithromycin, or ciprofloxacin (Wi et al., 2017). Gentamycin has been used as an alternative therapy in some places in Africa, although a few cases of resistance have been reported in other parts of the world (Chisholm et al., 2011). Whole-genome sequencing (WGS) allows for timely detection and elucidation of AMR determinants in bacteria (Collineau et al., 2019). High-throughput sequencing has many advantages, like long read lengths, short time, and reduction of bias introduced through amplification by PCR steps (Goldstein et al., 2019). WGS has been used to investigate quinolone-resistant gonorrhea outbreaks (Duncan et al., 2011; Kivata et al., 2019) in Kenya. It has also been used to analyze isolates resistant to ciprofloxacin, azithromycin, and ESCs in other countries (Chaudhry et al., 2002; Eyre et al., 2018; Golparian et al., 2018).

MinION, a mobile sequencing device from ONT (Oxford, United Kingdom), sequences DNA by monitoring the transfer of individual DNA molecules through various types of pores, resulting in very long and unbiased sequence reads, as there is no amplification or chemical reactions used during sequencing (Golparian et al., 2018). The mobile nature of the MinION offers many advantages and the sequencer has been evaluated for use in resource-limited settings and on the International Space Station (ISS) (Castro-Wallace et al., 2017; Elliott et al., 2020). MinION has also been shown to be effective for diagnostics, high-quality assemblies, and AMR surveillance in bacteria. However, the high error rate for the sequencer limits its use. Golparian and co-workers sequenced 14 clinical isolates of *N. gonorrhoeae* and evaluated multiple methods for genome assemblies using MinION with Illumina reads (Golparian et al., 2018). Street and co-workers used MinION to obtain a *de novo* assembly of *N. gonorrhoeae* isolated from patient urine samples (Street et al., 2020). Zhang and co-workers showed that AMR-profiling results for *N. gonorrhoeae* were comparable when using only MinION-based assemblies or MinION-Illumina hybrid assemblies (Zhang et al., 2020). Naidenov and co-workers used Nanopore-only sequencing to obtain complete genomes and carried out AMR profiling of two new strains from the *Elizabethkingia* genus (Naidenov et al., 2019). Sanderson and co-workers used nanopore sequencing data and showed that it is possible to generate accurate consensus bacterial genomes from metagenomic sequencing data without using Illumina short reads (Sanderson et al., 2020).

In a previous study from Kenya, 22 multi-drug-resistant gonococcal isolates from heterosexual patients (2 females and 20 males) were sequenced using Illumina MiSeq for understanding *gyrA* and *parC* mutations in ciprofloxacin resistance (Kivata et al., 2019). Cehovin et al. sequenced 103 genomes from 73 patients from coastal Kenya, mainly men who had sex with men, using Illumina HiSeq and analyzed the plasmids conferring antibiotic resistance (Cehovin et al., 2018). These studies in Kenya have used the Illumina platform and mainly focused on isolates from male patients. We sequenced 10 isolates derived from women who visited STI clinics in Nairobi between 2012 and 2017, using only MinION sequencing in Kenya and evaluated methods for assembly and AMR profiling of genomes. We have also carried out phylogenies with previously deposited sequences in PubMLST, from Kenya, and other countries. Here, we assess the possibility of using MinION data alone for WGS, assembly, and comparative analysis. This study demonstrates the utility of the portable MinION sequencer for genome-based analysis in regular clinical setups.

## Materials and Methods

### Bacterial Isolates and Culture Conditions

*Neisseria gonorrhoeae* isolates were derived from high vaginal swabs collected between January 2012 and December 2017. The stocked organisms were stored at –70°C and retrieved and cultured on Modified Thayer–Martin agar. The plates were incubated at 37°C and 5% carbon dioxide (under raised CO_2_) as per the standard protocol.

Isolates were identified by Gram stain, Catalase test, Oxidase test, and carbohydrate-utilization studies of glucose, maltose, fructose, and sucrose, and confirmed by analytical profile index testing kit (API NH, bioMérieux). All gonococcus isolates were re-stocked in 20% glycerol-tryptic soy broth for long-term storage at –70°C for external quality control, sequencing, and molecular characterization. Standard *N. gonorrhoeae* isolates, i.e., WHO K, WHO P, WHO O, WHO R, and WHO M (Unemo et al., 2016), were used to test the media for viability and colonial characteristics. Phenotypic characterization was performed by characterizing lactamase production determined using nitrocefin solution. In the present study, clinical isolates from 10 patients were sequenced (Supplementary Table 1).

### Antibiotic Susceptibility Testing

Ciprofloxacin, spectinomycin, cefixime, ceftriaxone, gentamycin, and cefoxitin strips were used to set *E*-test according to the Clinical and Laboratory Standards Institute (CLSI). An antimicrobial gradient diffusion (*E*-test) method was used to determine the minimum inhibitory concentration (MIC) of *N. gonorrhoeae*, and interpretation of susceptibilities for selected antimicrobial agents was carried out as recommended by CLSI (Supplementary Table 2).

### Isolation of Genomic DNA

All the colonies from Thayer–Martin agar (37°C in a humidified 5% CO_2_ environment for 36 h) with the phenotypic characteristics of *N. gonorrhoeae* were picked up to create a culture suspension in 1X PBS. The genomic DNA was isolated using the Qiagen DNA isolation kit using the manufacturer’s specifications. Extracted DNA was purified using AMPure XP beads (Beckman Coulter, United Kingdom), eluted in 50 μl of nuclease-free water, and quantified using a QiaXpert (Qiagen). Pipetting was minimized to reduce shearing of the DNA prior to sequencing.

### ONT Library Preparation and MinION Sequencing

DNA library preparation for Nanopore sequencing was carried out using Ligation Kit SQK-LSK109 (ONT). Fragmented DNA was repaired and dA-tailed using the NEBNext FFPE DNA Repair Mix and NEBNext Ultra II End Repair/dA-Tailing Module (New England BioLabs). An individual barcode was added to dA-tailed DNA by using the barcoding extension kit EXP-NPB104 in accordance with the ONT protocols with NEB Blunt/TA Ligase Master Mix (New England BioLabs). Each barcoded DNA was pooled in equimolar amounts, and an adapter was attached using the NEBNext Quick Ligation Module (New England BioLabs). The MinION flow cell and reagents were shipped from Bangalore, India, to Nairobi at 4°C. The number of active pores was checked before loading. The samples were pooled using equimolar pooling, the library was loaded into the SpotON flow cell R9.5 (FLO-MIN106), and sequencing was carried out on MinKNOW using the 48-h script.

### Base-Calling, Read Trimming, and Processing

Guppy (v 3.2.2) (ONT) was used for base-calling of fast5 files. Reads with a mean q score (quality) greater than 7 and a read length greater than 500 bp were used and trimmed for adaptor sequences and barcodes using qcat (v1.1.0)^1^ from ONT. The files corresponding to each barcode (sample) were analyzed as follows.

### Assembly and Assessment

The selected raw reads were corrected, trimmed, and assembled using Canu (v1.8)^2^ using the parameters genome Size = 2.1 m and minReadLength = 500 (Koren et al., 2017). Minimap (v 2.17)^3^ and Miniasm (v0.3)^4^ were used for the self-mapping of the raw reads and the concatenation of the alignments to get the *de novo* assembly, respectively (Li, 2016). Two rounds of error correction of this draft assembly were carried out with Racon (v1.4.3)^5^ using raw reads (Vaser et al., 2017). The above assemblies were polished using the raw nanopore reads with Nanopolish (v0.11.1)^6^ (Loman et al., 2015).

Species identification was carried out using the Ribosomal Multilocus Sequence Typing rMLST (Jolley et al., 2012) approach from BIGSdb and PubMLST to identify the causative organism and co-infections (Jolley et al., 2018). We used the *de novo* assemblies for the above step. The database uses 53 genes encoding the bacterial ribosome protein subunits (*rps* genes) for species assignment. We also used blastn with 16S rRNA and 23S rRNA as the queries for species confirmation. The plasmid sequences were identified from the *de novo* assemblies. The plasmid sequences were used for detecting the AMR determinants.

We also carried out a guided genome mapping using reference genome *N. gonorrhoeae* FA1090 (NCBI:txid485). The trimmed raw reads were aligned to the reference genome using the bwa-mem option from bwa (v0.7.12)^7^ (Li and Durbin, 2010). Samtools (v1.9)^8^ was used for deriving the bam alignment file and the consensus genome (bcftools), after normalizing the indels and variant calling (Li et al., 2009). The commands samtools mpileup with the options *-Ou -f* and bcftools calls with the options *-mv -Oz --ploidy 1* were used for variant calling. The options bcftools *filter --IndelGap 5* and bcftools *consensus -H ‘‘A’’* were used for deriving the consensus genome. Mummer (v3.9.4)^9^ (Kurtz et al., 2004) and bedtools (v2.25.0)^10^ (Quinlan and Hall, 2010) were used for calculating the correspondence of the assemblies with the reference genome, genome coverage, and sequencing depth. The base positions with zero coverage were extracted using awk commands, and bedtools maskfasta option was used to derive the draft genomes; missing nucleotides were marked in the genome as “N.”

### Annotation

The gene prediction and annotation were carried out using the RAST server^11^ using the reference genome FA1090 for guiding the gene prediction (Overbeek et al., 2013).

### Genome-Based MLST Analysis

Gene profiles for sequence typing of *N. gonorrhoeae* were downloaded from different databases like NG-STAR (*N. gonorrhoeae* Sequence Typing for Antimicrobial Resistance)^12^ (Demczuk et al., 2017), PubMLST (Public databases for multi-locus sequence-typing)^13^ (Bennett et al., 2007), and NG-MAST (*N. gonorrhoeae* multi-antigen sequence typing)^14^ (Martin et al., 2004). NG-MAST uses two highly polymorphic loci, the outer-membrane porin (*por*) and transferrin binding protein β-subunit *tbpB*, for sequence typing. NG-STAR uses variants in seven antimicrobial resistance determinants (*penA*, *mtrR*, *porB*, *ponA*, *gyrA*, *parC*, and 23S rRNA) to three classes of antibiotics (cephalosporins, macrolides, and fluoroquinolones), and MLST (database PubMLST) uses seven housekeeping genes (*abcZ*, *adk*, *aroE*, *fumC*, *gdh*, *pdhC*, and *pgm*) for sequence typing. Profiles were created and blastn (*E*-value 10^−7^) from standalone BLAST+ ^15^ (Altschul, 2005) was used to assign the genes to the different alleles for MLST genes.

### Identification of Mutations Causing AMR

Around 85 mutations in 14 genes reported to be involved in AMR were checked for in the sequenced strains, including promoter mutations resulting in overexpression of efflux proteins (Unemo and Shafer, 2011). Additionally, we checked for the presence of four drug efflux pumps, two genes from conjugative plasmids, and plasmid-mediated AMR determinants (Supplementary File). Mutations were manually screened for, in the protein sequences identified using the RAST server in all the genomes. Sequencing depth at the corresponding gene loci was calculated from the results of the samtools depth command. We compared our results with that of three publicly available databases for AMR, CARD (Comprehensive Antibiotic Resistance Database), ARIBA (Antimicrobial Resistance Identification By Assembly) (Hunt et al., 2017), and Pathogenwatch (Balloux et al., 2018; Alcock et al., 2019). Resistance genes from plasmids were identified using blastn (*E*-value 10^−7^) against NCBI Bacterial Antimicrobial Resistance Reference Gene Database^16^ (NCBI Resource Coordinators, 2017).

### Homology Modeling

To understand the basis of antibiotic resistance, wild-type and mutant proteins implicated in AMR were modeled using templates from other bacteria. Coordinates of heteroatoms like antibiotics and DNA are present in the templates used. Hence, the heteroatom modeling module from Modeler (v9.23) (Sánchez and Sali, 2000) was used to build a multi-chain model with symmetry restraints. Clustal Omega^17^ was used for the alignment of query protein sequences with the template protein sequence, with manual correction of the alignment (Sievers and Higgins, 2014). The residues in different chains were separated using “/” and the symbol “.” was used to indicate the ligand molecules in the alignment.

### Multiple-Sequence Alignment and Phylogenetics

Phylogenetic tree analysis was carried out using 123 previously sequenced *N. gonorrhoeae* strains from Kenya and an additional 130 strains from different countries across the world, standard WHO strains, and the reference genome. The Genome Comparator module from Bacterial Isolate Genome Sequence Database (BIGSdb)^18^ was used to obtain the sequences and generate core genome alignments (cgMLST1649-) using the MAFFT module within BIGSdb (Jolley et al., 2018). We used a dataset of strains from different WHO geographical regions including strains from Kenya and WHO reference strains. The parameters used were minimum percentage identity 70%, minimum percentage alignment 50%, and blastn search to annotate loci in genomes with ≥50% loci (word size of 20). Incomplete loci were not used for pairwise comparison and pairwise missing loci and paralogous loci were excluded from the analysis. The *N. meningitidis* strain (PubMLST ID: 12672| 053442, cc4821) was included as an outgroup. The alignments were checked for redundancy in sequences of core genes; the sequences with 100% redundancy and the sequences missing all the gene loci analyzed were removed. Phylogenetic trees were derived based on Maximum likelihood approach using RAxML software (v8.2.12) using GTR + GAMMA substitution model with 1000 bootstraps (Kozlov et al., 2019). The best ML search trees were identified for both the datasets (only Kenyan strains and combined dataset of all 255 strains) annotated with details like WHO geographical region and antibiotic sensitivity from BIGSdb and Cehovin et al. (2018) using iTOL (Letunic and Bork, 2016).

The workflow for consensus genome used in the study has been illustrated in Supplementary Figure 1A, and the subsequent analysis workflow has been shown in Supplementary Figure 1B. The workflow for the *de novo* assemblies has been shown in Supplementary Figure 1C.

## Results

### Overview of Sequenced Data

We obtained between 534 and 2,387 Mb of reads from the MinION sequencing runs for each sample. The longest reads ranged from 156 to 406 kb with an average length of 1.5–2.7 kb (Table 1). We sequenced clinical isolates from 14 patients and obtained 12 *N. gonorrhoeae* genome sequences. Out of these, 10 genomes were near-complete with a good sequencing depth and were used for subsequent analysis.

**TABLE 1:** Read and assembly statistics.

**Strain IDs**	**Genome coverage (%)**	**Sequencing depth**	**# Mapped reads**
3	99.03	523.604	308,387
12	99	377.339	216,691
18	98.78	272.294	175,050
57	98.81	1255.388	2,272,143
61	98.97	63.726	43,553
81	99.59	86.3	182,347
100A	98.68	848.073	1,460,162
196	98.2	22.6	143,725
240	99.59	86.3	70,185
274	98.88	240.667	180,375
285	98.31	129.085	116,342
298	98.67	219.616	212,198

*The number of mapped reads for each genome was obtained using samtools flagstat and the genome assembly statistics were obtained using QUAST. The genome coverage was not affected by the number of reads or the sequencing depth. The genomes assembled from strains 81 and 196 had low sequencing depth and lower coverage in the genes surveyed in the study and were not used for further analysis. The strains 4 and 11 were identified to be *N. meningitidis* genomes from the ribosomal MLST analysis and were not analyzed further in the study (Supplementary Table 1).*

### Genome Assembly Statistics and Species Identification

Overview of the mappability of the reads from different strains to the reference genome has been depicted in Supplementary Figure 2. Assemblies were compared and selected based on the following criteria: the lowest number of mismatches, misassemblies, contigs, and the highest fraction of genome coverage using QUAST (Gurevich et al., 2013) (Supplementary Tables 3A–C). The length of the assemblies and the number of CDS identified were fairly the same across different methods. No plasmid or transposon gene insertions were observed in the genomes assembled *de novo* and the consensus genome. Similar to previous reports, we observed frameshifts with the *de novo* assemblies (Golparian et al., 2018). The consensus genome (FA1090 was used for read mapping) was used for further analysis as the genome coverage, number of complete genomic features, number of indels, and number of mismatches were better.

Among the 14 samples, 2 samples showed the presence of *N. meningitidis*, 9 samples showed the presence of *N. gonorrhoeae*, and 3 samples showed the presence of both *N. gonorrhoeae* and *M. osloensis* (Supplementary Table 1). *N. meningitidis* has been implicated in STI in recent years, while *M. osloensis* is also a commensal of the urogenital tract (Slack, 2010; Johnson et al., 2016).

Although high error rates have been reported with the use of the MinION sequencer, we have used long sequencing time (48 h) and obtained high depths of sequencing (63-1255X; Table 1). Previous studies have shown that sequencing using MiNION for longer time duration increases the sequencing output and the error rate can be minimized by using depth and consensus (Tyler et al., 2018; Plesivkova et al., 2019).

### Sequence Typing of the Strains

The alleles used in sequence typing (MLST, NG-MAST, and NG-STAR) were assigned to the genes from different strains, and we identified few novel alleles. The novel allele sequences have been deposited in the PubMLST database and the assigned allele numbers have been mentioned (Supplementary Tables 4A–C). The sequencing depth at each of these loci was found to be very high (>50–600) (Supplementary Table 4D). This is higher than the recommended depth for SNP detection using Nanopore sequencing by Sanderson and co-workers (20X) (Sanderson et al., 2020).

### Antibiotic Resistance and Mutations in AMR Genes

The MIC results with six different antibiotics have been provided in Table 2.

**TABLE 2:** MIC values for different antibiotics.

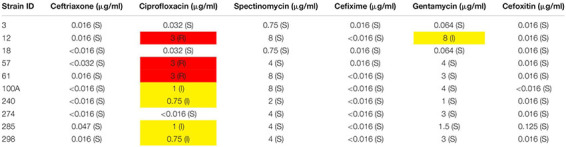

*The MIC values (μg/ml) and the interpretations for different antibiotics for the 10 strains. S – Susceptible, R – Resistant (highlighted in red), I – Intermediate (highlighted in yellow). The interpretation of MIC was inferred from CSLI breakpoints (Supplementary Table 2).*

A dataset of AMR targets was identified using literature (Supplementary Table 5A). These included mutations in the promoter region of repressors resulting in overexpression of efflux proteins, mutations in drug targets that result in higher MICs for the drugs either individually or in combination with other mutations, plasmids for drug resistance, and mosaic alleles in genes and promoter regions reported from drug-resistant strains of *N. gonorrhoeae* from across the world. Out of these, 28 mutations (in 11 genes) were observed in the strains (Table 3). The sequencing depth of *gyrA*, *parC*, *mtrR*, *ponA1*, *penA*, *porB*, *folP*, *rplD, pilQ, rpoB*, and *rpsJ* genes was high (Supplementary Tables 4D, 5B) and there were no missing bases in these genes. Other databases used for AMR profiling (Pathogenwatch, ARIBA, and CARD) missed a few of the mutations we identified (Supplementary Table 5C). The ARIBA database has not been updated; hence, it may be missing some recently identified AMR-associated mutations (Hunt et al., 2017). CARD collects AMR determinants from several bacteria and is not specific to *N. gonorrhoeae*; it may include mutations causing AMR in other bacteria but not gonococci (Alcock et al., 2019), whereas compared to these databases, Pathogenwatch is frequently updated; we identified the mutation *rplD* G70D (implicated in macrolide resistance) in three strains, but this mutation has been missed by Pathogenwatch (Ma et al., 2020b).

**TABLE 3:** AMR gene profile of the 10 strains.

		**Strains**										
		**Reference**	**3**	**12**	**18**	**57**	**61**	**100A**	**240**	**274**	**285**	**298**
**Gene**	**Mutation**											
***gyrA***	S91F/Y	S	S	**F**	**F**	**F**	S	**F**	**F**	S	**F**	S
	D95N/G/A	D	D	**A**	**A**	**A**	D	**A**	D	D	**A**	D
***parC***	E91K/G	E	E	**G**	E	E	E	E	**G**	E	**G**	**G**
***ponA1* (pbp1)**	L421P	L	**P**	L	**P**	**P**	L	**P**	**P**	L	**P**	L
***rpsJ***	V57M	V	**M**	**M**	**M**	**M**	**M**	**M**	**M**	**M**	**M**	**M**
***penA*/pbp2**	D345 insertion	–	**D**	**D**	**D**	**D**	**D**	**D**	**D**	**D**	**D**	**D**
	F504L	F	**L**	**L**	**L**	**L**	**L**	**L**	**L**	**L**	**L**	**L**
	A510V	A	**V**	**V**	**V**	**V**	**V**	**V**	**V**	**V**	**V**	**V**
	A516G	A	**G**	**G**	**G**	**G**	**G**	**G**	**G**	**G**	**G**	**G**
	P551S/L	P	P	P	**S**	**S**	P	**S**	**L**	**S**	**S**	**S**
***porB1b* (penb)**	G120D/K	G	G	G	**D**	**D**	G	**D**	**D**	G	G	G
	A121D	A	A	A	**D**	**G**	A	**G**	A	**S**	**S**	**G**
	N122	N	N	N	N	**D**	N	**S**	N	**K**	**K**	N
***pilQ* (penC)**	QAATPAKQ insertion at 180	–	–	–	–	–	–	–	–	**QAATPAK**	–	–
	D526N	D	D	D	D	D	D	D	D	D	**G**	D
	N640S	N	N	N	N	N	N	N	N	N	**S**	N
***folP***	T66M	T	**M**	T	T	T	T	T	**M**	T	**M**	T
	R228S	R	**S**	**S**	**S**	**S**	**S**	**S**	**S**	**S**	**S**	**S**
***mtrR***	A39T	A	**T**	**T**	**T**	**T**	**T**	**T**	A	A	A	A
	H105	H	H	H	H	H	H	H	**Y**	**Y**	**Y**	H
***rplD***	G70D/S/A/R	G	**D**	Y	G	Y	G	G	G	G	G	G
***rpoB***	H552N	H	**N**	H	**N**	**N**	H	**N**	H	H	H	H

Ciprofloxacin resistance		–	–	R	R	R	I	I	–	–	R	R
Gentamycin resistance		–	–	I	–	–	–	–	–	–	–	–

*Mutations observed in the studied strains have been listed, mutated residues are indicated in bold, and mutations identified in this study have been underlined. The resistance profile for the strains as inferred through MIC has been indicated for ciprofloxacin and gentamycin. The isolates were sensitive to the other antibiotics that were tested as inferred from Table 2. The newly identified mutations need to be experimentally investigated for their role in antibiotic resistance.*

We found six isolates carrying AMR mutations in penA C-terminal region (penicillin and cephalosporin resistance determinant) and four isolates with mutations in *ponA1* (penicillin and cephalosporin resistance determinant), respectively. We detected the D345 insertion and F504L mutation in *penA* (Zapun et al., 2016), which could be involved in penicillin and cephalosporin resistance (Table 3). Five isolates harbored *gyrA* resistance mutations, two of which also had a *parC* mutation. One strain had a mutation in only the *parC* gene; however, this is a silent mutation that was among the set of mutations observed in a cephalosporin-resistant isolate (Chaudhry et al., 2002). One of the ciprofloxacin-resistant strain contained a *gyrA* mutation, consistent with previous observations of these mutations in susceptible (Tanaka et al., 2000; Chaudhry et al., 2002) isolates. These mutations, in the Quinolone Resistance Determining Region (QRDR) of both the proteins, have been shown to contribute to the high MICs for ciprofloxacin (Belland et al., 1994). Interestingly, one of the *gyrA* mutations we observed, D95A/G, and the mutation E91G in ParC have been reported to be specific to Kenyan isolates (Kivata et al., 2019). Specific mutations in *porB1b* (penB AMR determinant), implicated in decreased influx of antimicrobials through the porin PorB (Lee et al., 2010; Chen and Seifert, 2013), were identified in 6 of the 10 isolates. All 10 strains harbored the V57M mutation in RpsJ, which has been shown to contribute to tetracycline resistance and R228S mutation in FolP, which results in sulfonamide resistance (Yun et al., 2012). Three strains harbored mutations in *rplD* gene, which is implicated in azithromycin resistance (Ma et al., 2020b). Eight strains had mutations in *pilQ* gene (PenC), implicated in penicillin, ESC, and tetracycline resistance, while four strains had the H522N mutation in *rpoB*, which is implicated in rifampicin resistance. Plasmid-mediated AMR determinants [high-level resistance to benzylpenicillin and tetracycline -p*bla*TEM and p*tet(M)*] were detected in all strains except one where we detected only the *tet(M)*-containing plasmid. We did not identify *mef* or *erm* containing conjugative plasmids/transposons, which are reported to cause resistance to macrolides (Roberts et al., 1999) or resistance-conferring mutations for spectinomycin, in either the *de novo* assemblies or consensus genome.

### Understanding the Basis of Antibiotic Resistance

We investigated the basis of drug resistance for the mutations in the proteins GyrA, ParC, FolP, porB1b, PonA1, and RpoB (Supplementary Figures 3–8, template details in Supplementary Table 6) through homology modeling using templates with co-crystallized antibiotic structures. The results have been described in Supplementary File (Section 8).

### Phylogenetic Relationship With Other *N. gonorrhoeae* Genomes From Kenya, Other WHO Geographical Regions, and WHO Strains

To understand the genetic relatedness of isolates from Kenya with isolates from other geographical regions and WHO reference isolates, we constructed a phylogenetic tree using the alignments derived from the core genome MLST (cgMLST). After filtering for sequence redundancy and loci completeness, we retained 238 sequences from different geographical regions across the world (Americas, Europe, Western Pacific, Africa and WHO reference strains) for the phylogenetic analysis. The metadata for the strains like geographical region and antibiotic resistance details have been indicated in the phylogenetic trees (details in Supplementary Table 7).

**FIGURE 1:**
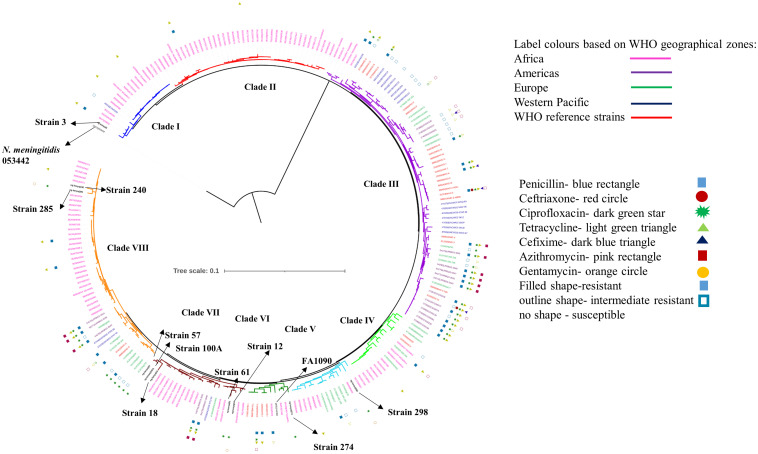
Phylogenetic tree of the sequenced strains with strains from WHO geographical regions and WHO reference strains. The strains sequenced in this study have been indicated in black, bold text in the tree. The antibiotic resistance profile, wherever available, has been indicated as metadata (Penicillin – blue rectangle, Ceftriaxone – red circle, Ciprofloxacin – dark green star, Tetracycline – right-pointing light green triangle, Cefixime – left-pointing dark blue triangle, Azithromycin – pink rectangle, Gentamycin – orange circle, Filled shape – resistant, outline shape – intermediate resistant, no shape – susceptible). The clades inferred from the phylogeny analysis have been indicated in different colors and numbered I–VIII. The strains from different WHO geographical regions have been indicated in different color labels (Africa – Pink, Americas – Magenta, Europe – Green, Western Pacific – Blue, WHO reference strains – Red) The strains sequenced in this study, the reference strain for *N. gonorrhoeae* (FA1090), and an *N. meningitidis* strain (PubMLST id:053442) used as an outgroup for the analysis have been labeled and indicated with arrows.

**FIGURE 2:**
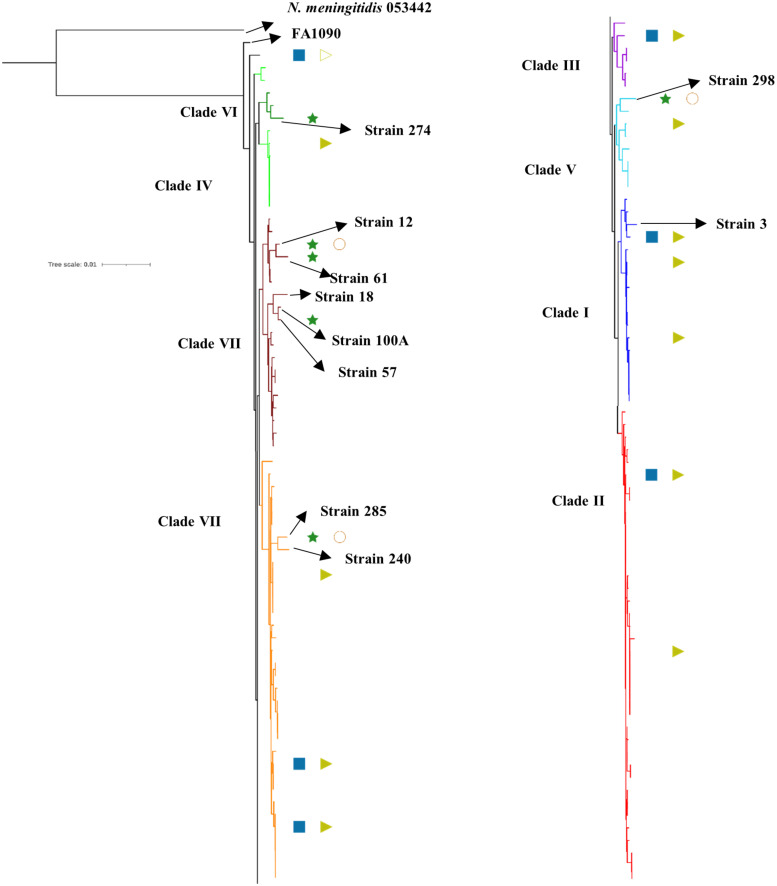
Phylogenetic tree of the sequenced strains with previously reported strains from Kenya. The antibiotic resistance profile, wherever available, has been indicated as metadata (Penicillin – blue rectangle, Ciprofloxacin – dark green star, Tetracycline – right-pointing light green triangle, Gentamycin – orange circle, Filled shape – resistant, outline shape – intermediate resistant, no shape – susceptible). The clades inferred from the phylogeny analysis have been indicated in different colors and numbered I–VIII as per Figure 1. The strains sequenced in this study, the reference strain for *N. gonorrhoeae* (FA1090), and an *N. meningitidis* strain (PubMLST id:053442) used as an outgroup for the analysis have been labeled and indicated with arrows.

We observed eight clusters of sequences from across the world (clusters I–VIII). Clades I and II consisted of only Kenyan sequences, while sequences from different geographical regions and Kenya co-clustered in the rest of the clades (Figure 1). Cehovin et al. had observed in an earlier study that strains from Kenya were distinct from strains sequenced from USA and the UK (Cehovin et al., 2018). Consistent with this observation, we see Kenyan sequence-specific clades, but we also observe clades where sequences from Kenya are mixed from sequences from other geographical regions. The clades were more or less consistent in the phylogenetic tree constructed using sequences from all over the world and using sequences from only Kenya (Figure 2). Out of the strains sequenced in the study, strain 3 clustered in clade I; strain 298 in clade V; clade 274 in clade VI; strains 12, 18, 61, 57, and 100A in clade VII; and strains 240 and 285 in clade VIII. Each cluster consisted of strains belonging to different multi-locus sequence types (MLSTs). Strains belonging to certain STs were specific to a cluster (ST1588 – cluster VII, ST1893 – cluster II) while strains from some other STs were found in multiple clusters (ST1599 in cluster III, IV, V, and VIII and ST1902 in clusters III and VIII) (metadata in Supplementary Table 7). Broader sampling and analysis of clinical isolates from Kenya can help us determine the extent of genetic diversity within the *N. gonorrhoeae* strains.

## Discussion

In this study, we assessed the feasibility of nanopore sequencing and AMR gene annotation in a clinical microbiology laboratory setting. We found that DNA extraction followed by MinION sequencing facilitates efficient detection of chromosomal and plasmid-mediated AMR genes in clinical isolates. From the 14 isolates sequenced in this work, we obtained 12 *N. gonorrhoeae* genomes, out of which 10 had more than 98% coverage with high depth in the AMR and MLST gene regions analyzed in the study. We were able to detect mutations in multiple genes involved in AMR and we also focused on investigating the structure–function aspects of drug resistance mutations in some proteins.

The consensus genome mapping obtained using bwa-mem was chosen for analysis, due to higher genome coverage and lower number of indels and misassemblies compared to the reference genome. Although earlier studies have shown that bacterial MinION sequences show high mapping rates to the reference genome, with fewer indel rates, bwa-mem has found limited application with long error-prone reads (Finn et al., 2014; Laver et al., 2015; Gerstmans et al., 2020; Nguyen et al., 2020). We show that it is possible to obtain assemblies with good genome coverage for AMR determinant detection by using nanopore-only reads with a consensus genome mapping strategy. Previous studies have obtained hybrid genome assembly for gonococcal isolates by combining short-read (Illumina) and long-read (ONT) sequence data (Eyre et al., 2018; Golparian et al., 2018). This is another approach for deriving a consensus genome using MinION sequencing and, accordingly, an alternative to *de novo* assembly.

Although the error rate for single nanopore reads is higher, consensus approaches can result in highly reliable assemblies. Assemblies derived from MinION-1D sequencing have shown a good agreement between AMR testing and AMR prediction results (Lemon et al., 2017; Sanderson et al., 2020). Studies on comparison of assemblies derived from Nanopore-only, Illumina, and hybrid assemblies have shown that the AMR prediction is comparable across all three methods (Chen et al., 2020). Recent advances in Nanopore sequencing and analysis workflows have demonstrated the utility of ONT sequencers for metagenomic analysis (Sanderson et al., 2020; Street et al., 2020). Additionally, there are workflows available for real-time sequence analysis that make the process faster.

Databases like PubMLST, CARD, ARIBA, and Pathogenwatch include profiles for resistance mediators like *penA*, *mtrR*, *penB*, *ponA*, 23S rDNA, *gyrA*, and *tetM* and do not include other determinants in their gonococcal AMR characterization module. We identified a set of AMR determinants from literature, including chromosomal gene mutations, promoter mutations, and the presence of plasmids with AMR genes. Plasmids containing genes encoding beta-lactamase and tetracycline resistance mediators [*tet*(M) and *bla*TEM] were identified in all but one strain (strain 12 contained only a TetM containing plasmid). This is consistent with a previous study that the plasmids are almost ubiquitous in Kenyan strains, thought to be because of the overuse of doxycycline for treating STIs (Cehovin et al., 2018). Among the reported mutations, we observed S91F and D95Y in the QRDR (residues 55–110) of GyrA and E91G in the ParC QRDR (residues 66–119). We also observed the silent mutations Y104 (codon change TAC − > TAT seen in all isolates except 298) and L131 (codon change of CTG − > CTA seen in isolates 3 and 240) in ParC reported to be associated with ciprofloxacin-resistant strains (Chaudhry et al., 2002). Novel mutations, V68A in the QRDR region of ParC (strain 285), E79 insertion in FolP (all isolates), and G70Y in RplD (strains 12 and 57), were also observed. However, whether these mutations have any effect on antibiotic resistance has to be experimentally validated. We were able to explain the basis of ciprofloxacin resistance in all strains except one, strain 298, which showed no mutations in gyrA, but had the E91G mutation in parC. We did not identify plasmids containing aminoglycoside resistance determinants like aminoglycoside N-acetyltransferases (*aat*) for gentamycin resistance (Garneau-Tsodikova and Labby, 2016).

Based on homology modeling using templates crystalized with antibiotics, we see that the mutations in GyrA disrupt the hydrogen bonding with ciprofloxacin, and mutations in ParC occur very close to Ser 88, which is involved in hydrogen bonding with moxifloxacin. We also observe that mutations in FolP at the substrate-binding site could affect the interaction with the antibiotic, as the residue at R228 interacts with sulfonamide. A phylogenetic study with previously sequenced Kenyan strains clustered our strains in the previously described cluster. From phylogenetic analysis using sequences from different countries, we observed five major clusters, with most of the Kenyan sequences (including the sequences from this study) clustered together with strains from different geographical regions.

We anticipate that the nanopore sequencing technology will expand the scope for rapid genome sequencing, which can help us understand the mechanistic basis of drug resistance and clinical management. AMR in *N. gonorrhoeae* is a result of a single to multiple mutations in same or different genes acting synergistally and additional unknown mutations/genes may be involved (Harrison et al., 2016; Ma et al., 2020a). Hence, there is a need for updated resistance gene databases and more studies on AMR genotype–phenotype correlations (Lemon et al., 2017). Recent advances have also shown promise for identifying clinical isolates and detecting AMR elements in real time (Schmidt et al., 2017; Sanderson et al., 2018). However, the read error rates mandate a consensus approach for read assembly and AMR allele detection. With future improvements in read accuracy and computational tools for base-calling, we believe that AMR detection can happen in clinical settings within hours of sequencing (Lemon et al., 2017).

## Conclusion

Using a literature mining approach, we have predicted ciprofloxacin resistance using mutations in *gyrA*/*parC* in strains showing decreased susceptibility to ciprofloxacin. However, few of the *gyrA* mutations can also occur in susceptible strains, and molecular tests using gene-based PCR or WGS can be used to complement culture-based antibiotic resistance testing. Culture-based testing can reflect mutations in unknown antibiotic targets, but cannot predict if a patient can develop resistance later on, based on pre-existing mutations.

In conclusion, in the first consensus genome for gonococci using only MinION, we show that using this approach, we can obtain near-complete genomes that were effectively used for AMR and phylogenetic analysis. We also show that currently available tools for AMR analysis of gonococci are not able to capture many mutations listed in the literature. We have also provided a dataset of over 100 existing mutations in different genes implicated in AMR, plasmids, and efflux pumps, which can be used by researchers across the world. This list will be continuously updated to keep up with the identification of new AMR targets/mutations.

Here, we demonstrate the potential of using MinION in resource-limited settings where NGS facility is unavailable. This can be used in settings with concerns about the export of samples/DNA for WGS to other countries. This approach can also be used across a range of bacterial genomes, even in case of metagenomic samples, where reads from a bacterial genome can be separated from one another, for subsequent analysis. Testing in clinical datasets can potentially provide a new approach to complement traditional methods for diagnosis, AMR surveillance, and public health management of *N. gonorrhoeae*. Further work is required in evaluating the MinION sequencer for outbreak prediction and clinical diagnosis of bacterial infection.

## Data Availability Statement

The raw reads generated for this study have been deposited in BioProject Accession: PRJNA660404 (https://www.ncbi.nlm.nih.gov). The biosample details are available under the IDs SAMN15960547, SAMN15960548, SAMN15960549, SAMN15960550, SAMN15960551, SAMN15960552, SAMN15960553, SAMN15960554, and SAMN15960555. The corresponding genomes and annotation files are available under the IDs CP061491, CP061490, CP061498, CP061488, CP061487, CP061486, CP061492, CP061485, CP061484, and CP061483.

## Ethics Statement

The ethics approval for the study was obtained from the Kenyatta National Hospital – University of Nairobi Ethics and Research Committee, Nairobi, Kenya, for the use of anonymized samples collected from female patients attending local Sexually Transmitted Disease (STD) clinics.

## Author Contributions

MJ collected the isolates and carried out characterization, culturing, and sequencing. AS, TD, AP, and RK planned and carried out the sequencing. IM conceived and performed the bioinformatics analysis, and wrote the manuscript with inputs from SK, AS, MM, and MJ. RN, PM, MM, RC, JN, and JG performed the experiments. SK, RS, and OA provided intellectual support and valuable discussions. All authors listed have made a substantial contribution to the work and approved it for publication.

## Conflict of Interest

The authors declare that the research was conducted in the absence of any commercial or financial relationships that could be construed as a potential conflict of interest.

## Publisher’s Note

All claims expressed in this article are solely those of the authors and do not necessarily represent those of their affiliated organizations, or those of the publisher, the editors and the reviewers. Any product that may be evaluated in this article, or claim that may be made by its manufacturer, is not guaranteed or endorsed by the publisher.

## Footnotes

1
https://github.com/nanoporetech/qcat

2
https://github.com/marbl/canu

3
https://github.com/lh3/minimap2

4
https://github.com/lh3/miniasm

5
https://github.com/isovic/racon

6
https://github.com/jts/nanopolish

7
http://bio-bwa.sourceforge.net/

8
http://www.htslib.org/

9
http://mummer.sourceforge.net/

10
https://bedtools.readthedocs.io/en/latest/

11
https://rast.nmpdr.org/

12
https://ngstar.canada.ca/

13
https://pubmlst.org/

14
http://www.ng-mast.net/

15
https://ftp.ncbi.nlm.nih.gov/blast/executables/blast+/

16
https://www.ncbi.nlm.nih.gov/bioproject/PRJNA313047/

17
https://www.ebi.ac.uk/Tools/msa/clustalo/

18
https://pubmlst.org/software/database/bigsdb/
