# Supplementary material for: Antimicrobial Resistance Profiling and Phylogenetic Analysis of Neisseria gonorrhoeae Clinical Isolates From Kenya in a Resource-Limited Setting
Source: Front Microbiol. 2021 Jul 27;12:647565. doi: 10.3389/fmicb.2021.647565 (PMC8353456; doi:10.3389/fmicb.2021.647565)
Supplement: Supplementary file 1 [file Data_Sheet_1.docx]

**Supplementary File**

**Antimicrobial resistance profiling and phylogenetic analysis of *Neisseria gonorrhoeae* clinical isolates from Kenya in a resource-limited setting**

Meshack Juma^1+^, Arun Sankaradoss^2+^, Redcliff Ndombi^1^, Patrick Mwaura^1^, Tina Damodar^2^, Junaid Nazir^2^, Awadhesh Pandit^2^, Rupsy Khurana^2^, Moses Masika^1^, Ruth Chirchir^1^, John Gachie^1^, Sudhir Krishna^2,3^, Ramanathan Sowdhamini^2^, Omu Anzala^1,*^, Iyer Meenakshi S^2,*^

^+^ These authors share first authorship

^*^These authors share corresponding authorship (Iyer Meenakshi S: meenakshis@ncbs.res.in, Omu Anzala: oanzala@kaviuon.org)

1 KAVI Institute of Clinical Research, University of Nairobi, Nairobi, Kenya

2 National Centre for Biological Sciences, TIFR, Bangalore, Karnataka, India

3 School of Interdisciplinary Life Sciences, Indian institute of Technology, Goa

1. **Sample details**

**Supplementary Table 1: Demographic information of patients.** Samples were taken with consent from >18 years old patients visiting STI clinics with genital discharge or fluid (watery, pus). CSW- commercial sex work, NG- *N. gonorrhoeae*, NM- *N. meningitidis*, MO- *Moraxella osloensis*, HIV- Human immunodeficiency virus. Only NG genomes were analysed further, genomes marked with * were found to have a lot of gaps and were not considered. R – Radio, B- Barazas, T- Television, F – Friends, N- Newspaper, O- Other sources.

| **ID** | **Enrol_No** | **District** | **CSW Duration Years** | **Clients Past Week** | **HIV Info Source** | **Education level** | **Services expected** | **Reads from organism** |
| --- | --- | --- | --- | --- | --- | --- | --- | --- |
| **3** | SW0002 | Murang'a | 1 | 0 | R, B | 2 | Treatment and doctor examination | NG |
| **4** | SW0004 | Kiambu | 11 | 0 | R | 2 | Doctor examination, HIV testing and treatment | NM |
| **11** | SW0280 | Nyandarua | 4 | 1 | N,R,T,F | 1 | HIV test and STI screening | NM |
| **12** | SW0005 | Murang'a | 2 | 0 | R | 2 | HIV test | NG |
| **18** | SW0018 | Machakos | 20 | 1 | B | 1 | Doctor examination | NG |
| **57** | SW0011 | Murang'a | 5 | 0 | T, F | 0 | HIV testing | NG |
| **61** | SW0032 | Nairobi | 4 | 2 | O | 2 | Doctor examination | NG |
| **81** | SW0053 | Murang'a | 4 | 1 | R | 1 | Doctor examination | NG* + MO |
| **100A** | SW0015 | Kiambu | 1 | 0 | F | 2 | HIV test and STI screening | NG |
| **196** | SW0102 | Kiambu | 6 | 1 | F | 1 | Doctor examination | NG* + MO |
| **240** | SW0223 | Thika | 6 | 1 | F | 1 | Doctor examination | NG + MO |
| **274** | SW0236 | Gem | 1 | 1 | R | 1 | Doctor examination | NG |
| **285** | SW0259 | Murang'a | 5 | 2 | R | 1 | Doctor examination | NG |
| **298** | SW0274 | Murang'a | 10 | 1 | F | 1 | Doctor examination | NG |

1. **Determining antibiotic susceptibility**

1. E-test strips were allowed to come to room temperature before opening the container.

2. Using the Vitek colourimeter or standard turbidity tube, we prepared a suspension of the test organism in sterile Muller Hinton broth, the organism was inoculated until it reached sufficient turbidity equivalent to 0.5 McFarland standards.

1. Dacron swab was used to inoculate from the pure culture onto an agar plate, streaking in 3 directions i.e. over the entire agar surface.
2. Incubation was carried out at 37°C and CO_2_ concentration (5%) and interpreted as per the instruction on the insert.

MIC’s above a documented specific level for each antibiotic as per the CLSI guidelines constitute “resistance” of a strain to that antibiotic while MIC’s above the normal MIC level but below the critical level for the definition of resistance constitute strains with decreased susceptibility to a given antibiotic.

**Supplementary Table 2:** **MIC reference used in the study.** MICs (µg/ml) for the interpretation of susceptibility of strains of *N. gonorrhoeae* to selected antimicrobial agents, as recommended by CLSI for interpretation of resistance.

| **Antimicrobial agents** | **Susceptible (S)** | **Intermediate (I)** | **Resistant (R)** |
| --- | --- | --- | --- |
| Ceftriaxone | ≤0.25 µg/ml | . | >0.25 µg/ml |
| Cefixime | ≤0.25 µg/ml | . | >0.25 µg/ml |
| Ciprofloxacin | ≤0.06 µg/ml | 0.12 – 0.5 µg/ml | >1 µg/ml |
| Spectinomycin | ≤ 32 µg/ml | 64 µg/ml | >128 µg/ml |
| Gentamycin | ≤ 4 µg/ml | 8 – 16µg/ml | >32 µg/ml |
| Cefoxitin | ≤ 2 µg/ml | 4 - 8 µg/ml | >8 µg/ml |

1. **Genome assembly and read statistics**

**
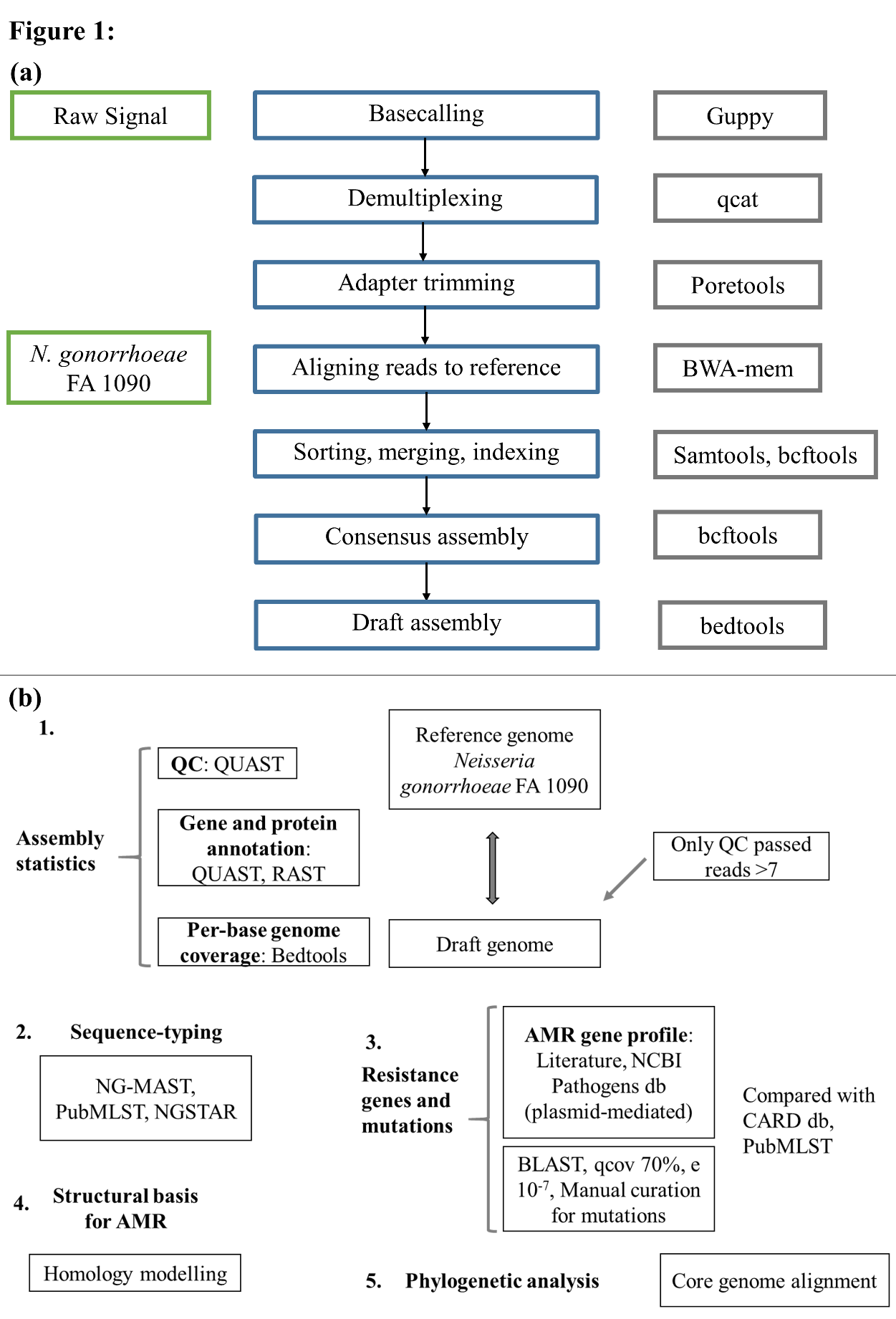
**

**Supplementary Figure 1a-b: The work-flow for deriving the consensus genome (a) and downstream analysis (b) of the *N. gonorrhoeae* genomes analysed in the study.**

We derived a consensus genome using bwa-mem and used RAST for genome annotation. For sequence-typing, we carried out BLAST searches with alleles derived from PubMLST and a set of AMR determinants was identified through literature and was used for screening the clinical isolates. Structure modelling of the proteins with mutations was carried out using templates with crystallised antibiotic structures to understand the basis of drug resistance.

**(c)**


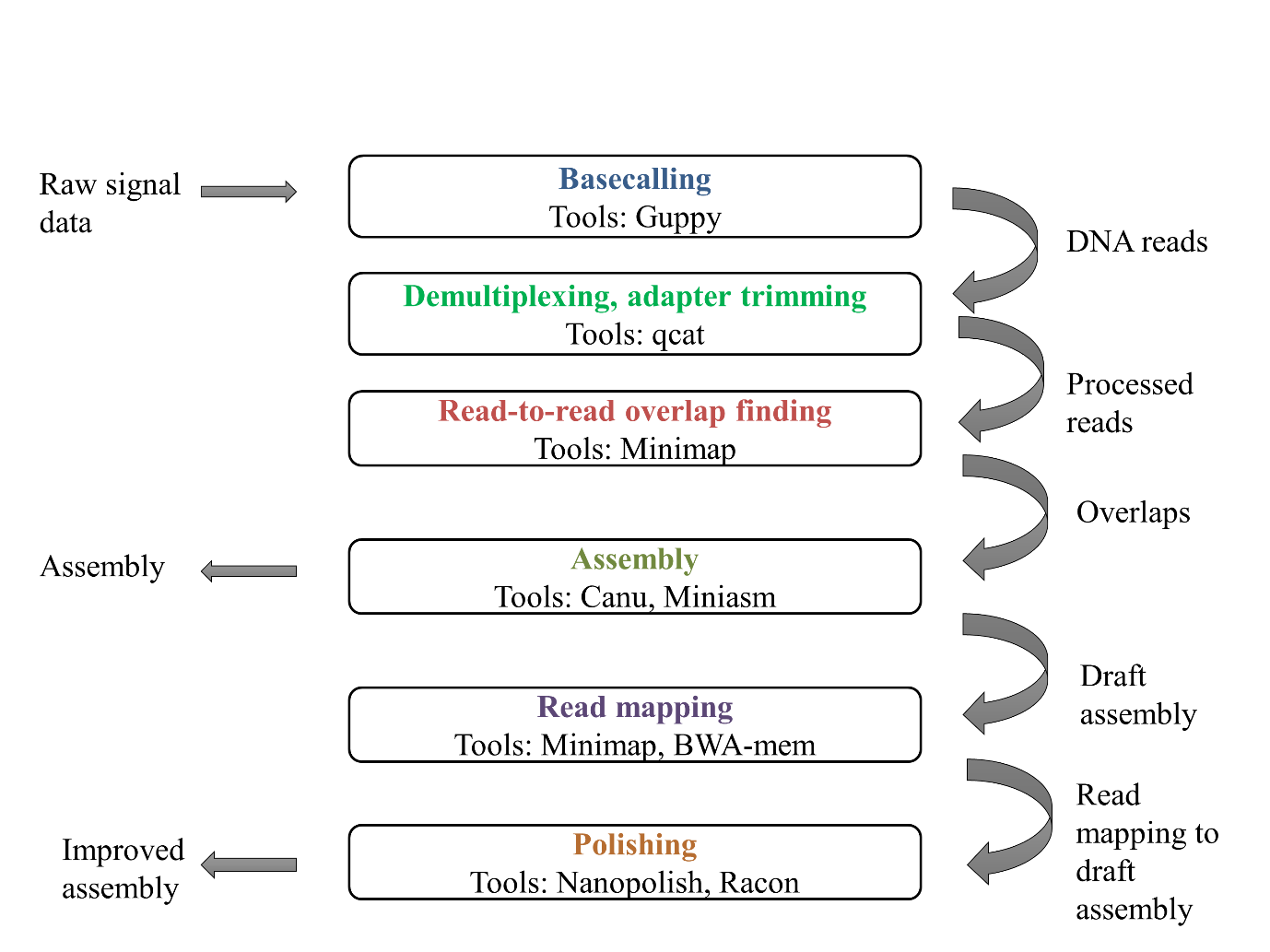


**Supplementary Figure 1c: Pipeline for *de novo* assembly.** The workflow followed for *de novo* genome assembly, error correction and genome polishing using Canu^1^ and Minimap-Miniasm^2^, has been depicted in the figure.

**
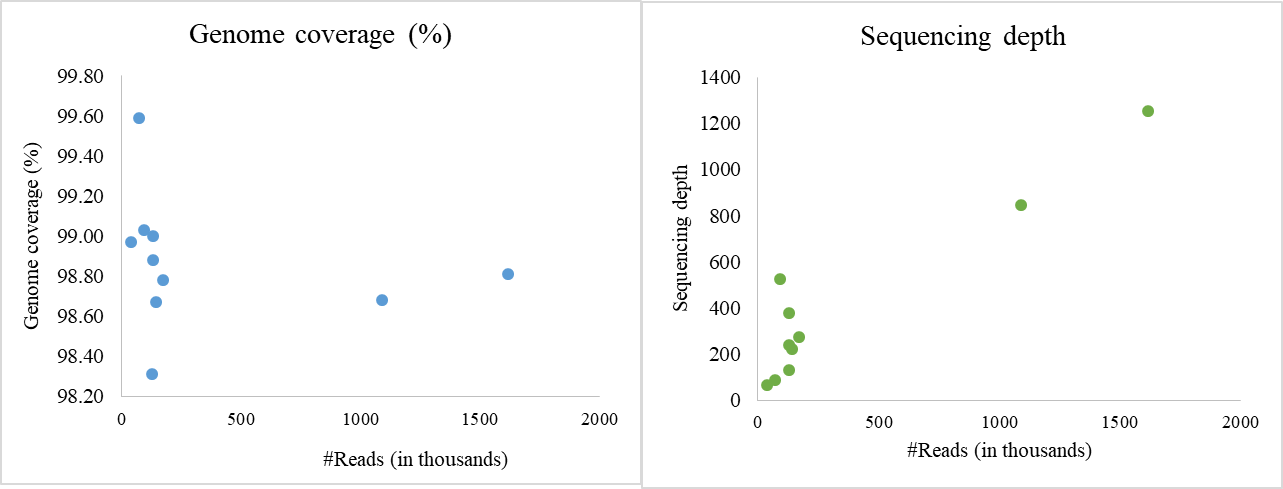
**

**Supplementary Figure 2: Mappability of the reads to the reference genome FA1090.** The number of reads vs depth and genome coverage for the *N. gonorrhoeae* strains has been plotted. Sequencing depth was calculated from the results of the samtools depth command. We observe that the increase in reads/coverage does not necessarily indicate an increase in genome coverage.

1. **Comparison of assembly statistics for assemblies obtained using different methods**

**Supplementary Table 3a: Assembly statistics for the consensus genome.** Bwa-mem^3^ was used for the assembly followed by samtools^4^ and bcftools for deriving the consensus from the assembly. The number of complete genomic features identified using QUAST^5^ webserver has been provided and the number of partial features has been indicated in brackets.

| **Strain ids.** | | | | | | | | | | |
| --- | --- | --- | --- | --- | --- | --- | --- | --- | --- | --- |
|  | **3** | **12** | **18** | **57** | **61** | **100A** | **200** | **274** | **285** | **298** |
| **Genome coverage (%)** | 99.677 | 99.772 | 99.654 | 99.87 | 99.867 | 99.793 | 97.83 | 99.386 | 99.839 | 99.675 |
| **Duplication ratio** | 1 | 1 | 0.998 | 1 | 0.999 | 1 | 1 | 0.999 | 0.999 | 0.998 |
| **No. of genomic features** | 1944 + (5) | 1950 + (4) | 1927 + (27) | 1949 + (5) | 1944 + (10) | 1950 + (4) | 1946 + (8) | 1921 + (33) | 1942 + (12) | 1936 + (18) |
| **Largest alignment** | 2146853 | 2148040 | 2142564 | 2150646 | 2149468 | 2149055 | 2150731 | 2136056 | 2148248 | 2142953 |
| **Total aligned length** | 2146853 | 2148040 | 2142564 | 2150646 | 2149468 | 2149055 | 2150731 | 2136056 | 2148248 | 2142953 |
| **NGA50** | 2146853 | 2148040 | 2142564 | 2150646 | 2149468 | 2149055 | 2150731 | 2136056 | 2148248 | 2142953 |
| **LGA50** | 1 | 1 | 1 | 1 | 1 | 1 | 1 | 1 | 1 | 1 |
| **No. of misassemblies** | 0 | 0 | 0 | 0 | 0 | 0 | 0 | 0 | 0 | 0 |
| **Misassembled contigs length** | 0 | 0 | 0 | 0 | 0 | 0 | 0 | 0 | 0 | 0 |
| **No. of mismatches per 100 kbp** | 594.93 | 579.1 | 659.97 | 628.56 | 583.71 | 616.15 | 654.45 | 602.37 | 556.95 | 616.04 |
| **No. of indels per 100 kbp** | 2.38 | 3.4 | 5.12 | 3.16 | 3.44 | 3.16 | 5.53 | 9.48 | 6.05 | 10.29 |

**Supplementary Table 3b: Assembly statistics for the *de novo* assembly derived from Minimap and Miniasm.** Error correction was done for two rounds with Racon, followed by polishing with Nanopolish to get the final assembly. The number of complete genomic features identified using QUAST webserver has been provided and the number of partial features has been indicated in brackets.

| **Strain ids** | | | | | | | | | | |
| --- | --- | --- | --- | --- | --- | --- | --- | --- | --- | --- |
|  | **3** | **12** | **18** | **57** | **61** | **100A** | **240** | **298** | **285** | **274** |
| **Genome coverage (%)** | 97.58 | 97.662 | 97.422 | 97.236 | 97.499 | 97.21 | 96.207 | 96.965 | 96.441 | 51.891 |
| **Duplication ratio** | 1.166 | 1.008 | 1.009 | 1.009 | 1.005 | 1.053 | 1.02 | 1.011 | 1.019 | 1.015 |
| **No. of genomic features** | 1886 + (36) | 1878 + (48) | 1869 + (56) | 1871 + (48) | 1881 + (41) | 1883 + (35) | 1855 + (37) | 1877 + (37) | 1872 + (36) | 438 + (894) |
| **Largest alignment** | 287696 | 342226 | 343711 | 328276 | 249880 | 339433 | 269000 | 360246 | 340123 | 41545 |
| **Total aligned length** | 2437622 | 2104585 | 2111549 | 2098835 | 2110445 | 2187640 | 2095088 | 2097842 | 2112454 | 1134422 |
| **NGA50** | 153182 | 239934 | 174316 | 199980 | 232383 | 197883 | 189013 | 194904 | 195276 | 1988 |
| **LGA50** | 5 | 4 | 5 | 5 | 5 | 5 | 5 | 5 | 5 | 94 |
| **No. of misassemblies** | 33 | 21 | 31 | 28 | 22 | 34 | 36 | 33 | 30 | 150 |
| **Misassembled contigs length** | 2177529 | 2171190 | 2164091 | 2163870 | 2159924 | 2264029 | 2223993 | 2218792 | 2250396 | 2175989 |
| **No. of mismatches per 100 kbp** | 1038.49 | 830.83 | 898.02 | 842.11 | 852.36 | 886.75 | 917.56 | 874.14 | 859.3 | 4569.89 |
| **No. of indels per 100 kbp** | 563.37 | 453.76 | 331.82 | 485.44 | 380.52 | 509.16 | 447.87 | 397.79 | 376.99 | 306.62 |

**Supplementary Table 3c: Assembly statistics for the *de novo* assembly derived from Canu.** Error-correction and trimming were done using Canu followed by genome polishing with Nanopolish. The number of complete genomic features identified using QUAST webserver been provided and the number of partial features has been indicated in brackets.

| **Strain ids.** | | | | | | | | | | |
| --- | --- | --- | --- | --- | --- | --- | --- | --- | --- | --- |
|  | **3** | **12** | **18** | **57** | **61** | **100A** | **240** | **298** | **285** | **274** |
| **Genome coverage (%)** | 97.58 | 97.662 | 97.422 | 97.236 | 97.499 | 97.21 | 96.207 | 96.965 | 96.441 | 51.891 |
| **Duplication ratio** | 1.166 | 1.008 | 1.009 | 1.009 | 1.005 | 1.053 | 1.014 | 1.011 | 1.019 | 1.015 |
| **No. of genomic features** | 1886 + (36) | 1878 + (48) | 1869 + (56) | 1871 + (48) | 1881 + (41) | 1883 + (35) | 1873 + (40) | 1877 + (37) | 1872 + (36) | 438 + (894) |
| **Largest alignment** | 287696 | 342226 | 343711 | 328276 | 249880 | 339433 | 334358 | 360246 | 340123 | 41545 |
| **Total aligned length** | 2437622 | 2104585 | 2111549 | 2098835 | 2110445 | 2187640 | 2113499 | 2097842 | 2112454 | 1134422 |
| **NGA50** | 153182 | 239934 | 174316 | 199980 | 232383 | 197883 | 195256 | 194904 | 195276 | 1988 |
| **LGA50** | 5 | 4 | 5 | 5 | 5 | 5 | 5 | 5 | 5 | 94 |
| **No. of misassemblies** | 33 | 21 | 31 | 28 | 22 | 34 | 35 | 33 | 30 | 150 |
| **Misassembled contigs length** | 2177529 | 2171190 | 2164091 | 2163870 | 2159924 | 2264029 | 2238288 | 2218792 | 2250396 | 2175989 |
| **No. of mismatches per 100 kbp** | 1038.49 | 830.83 | 898.02 | 842.11 | 852.36 | 886.75 | 891.39 | 874.14 | 859.3 | 4569.89 |
| **No. of indels per 100 kbp** | 563.37 | 453.76 | 331.82 | 485.44 | 380.52 | 509.16 | 407.19 | 397.79 | 376.99 | 306.62 |

1. **Sequence-typing analysis for the strains**

**Supplementary Table 4a: Sequence-typing results of the *N. gonorrhoeae* strains using the MLST**^6^ **scheme.** We observed novel alleles for the gene *pgm* in all the strains, *pdhC* in six strains, *adk* in four strains and *aroE* in two strains. The allele numbers assigned by PubMLST^7^ have been indicated in brackets. N: Novel allele.

| **Strain ids** | ***abcZ*** | ***adk*** | ***aroE*** | ***fumC*** | ***gdh*** | ***pdhC*** | ***pgm*** | **Sequence type** |
| --- | --- | --- | --- | --- | --- | --- | --- | --- |
| **3** | 59 | 67 | 67 | 111 | 148 | N (1104) | N (1181) | 15895 |
| **12** | 59 | N (848) | N (1127) | 158 | 148 | N (1105) | N (1182) | 15896 |
| **18** | 59 | 39 | N (1126) | 158 | 148 | N (1103) | N (1179) | 15893 |
| **57** | 59 | 112 | 67 | 158 | 148 | 71 | N (1180) | 15897 |
| **61** | 59 | 39 | 170 | 158 | 148 | N (1103) | N (1180) | 15894 |
| **100A** | 59 | N (849) | 67 | 158 | 148 | 71 | N (1183) | 15898 |
| **240** | 126 | N (848) | 67 | 157 | 148 | N (1107) | N (1182) | 15899 |
| **274** | 59 | 39 | 170 | 237 | 148 | N (1104) | N (1182) | 15900 |
| **285** | 126 | N (850) | 806 | 158 | 148 | 783 | N (1181) | 15901 |
| **298** | 59 | N (848) | 806 | 157 | 150 | N (1106) | N (1184) | 15902 |

**Supplementary Table 4b: Sequence-typing results of the *N. gonorrhoeae* strains using NG-MAST**^8^ **scheme.** Novel *por* and *tbpB* alleles were observed in strains except for strain 3 where the porin allele matched POR139. N: Novel allele.

| **Strain ids.** | ***por*** | ***tbpB*** |
| --- | --- | --- |
| **3** | POR139 | N (2961) |
| **12** | N (11470) | N (2962) |
| **18** | N (11466) | N (2963) |
| **57** | N (11469) | N (2964) |
| **61** | N (11471) | N (2965) |
| **100A** | N (11468) | N (2966) |
| **240** | N (11465) | N (2967) |
| **274** | N (11473) | N (2968) |
| **285** | N (11472) | N (2969) |
| **298** | N (11467) | N (2970) |

**Supplementary Table 4c: Sequence-typing results of the *N. gonorrhoeae* strains using NG-STAR**^9^ **scheme.** New alleles for *penA* were observed in all the strains, new *mtrR* alleles were observed in seven strains and new *parC* alleles were observed in two strains. The allele numbers assigned by PubMLST have been indicated in brackets. N: Novel allele.

| **Strain ids.** | ***penA*** | ***mtrR*** | ***porB*** | ***ponA*** | ***gyrA*** | ***parC*** | **23S rDNA** | **Sequence Type** |
| --- | --- | --- | --- | --- | --- | --- | --- | --- |
| **3** | N (193.001) | 10 | 100 | 1 | 100 | 2 | 100 | 3854 |
| **12** | N (194.001) | N (378) | 100 | 100 | 7 | 49 | 100 | 3857 |
| **18** | N (14.018) | N (377) | N | 1 | 7 | N (148) | 100 |  |
| **57** | N (14.019) | N (379) | N (66) | 1 | 7 | 7 | 100 |  |
| **61** | N (192.001) | 54 | 100 | 1 | 100 | 1 | 100 | 3856 |
| **100A** | N (14.017) | N (379) | N | 1 | 7 | 7 | 100 |  |
| **240** | N (190.001) | 69 | N | 100 | N (50) | N (174) | 100 |  |
| **274** | N (191.001) | N (380) | 3 | 100 | 100 | 2 | 100 | 3855 |
| **285** | N (9.01) | N (381) | 3 | 1 | 5 | N (149) | 100 |  |
| **298** | 1.002 | N (382) | 3 | 100 | 100 | 49 | 100 | 3858 |

**Supplementary Table 4d: Sequencing depth for the gene loci in PubMLST, NG-MAST and NG-STAR scheme.** The sequencing depth for the genic loci listed in (a)-(c) has been provided.

| **Strain id** | | | | | | | | | | | |
| --- | --- | --- | --- | --- | --- | --- | --- | --- | --- | --- | --- |
|  |  | **3** | **12** | **18** | **57** | **61** | **100A** | **240** | **274** | **285** | **298** |
| **ST scheme** | **gene** |  |  |  |  |  |  |  |  |  |  |
| **MLST** | ***abcZ*** | 182 | 340 | 189 | 310 | 40 | 768 | 53 | 193 | 84 | 168 |
|  | ***adk*** | 541 | 352 | 211 | 362 | 52 | 827 | 48 | 209 | 70 | 205 |
|  | ***aroE*** | 606 | 367 | 222 | 404 | 56 | 915 | 47 | 186 | 84 | 178 |
|  | ***fumC*** | 525 | 333 | 193 | 368 | 38 | 877 | 43 | 193 | 79 | 191 |
|  | ***gdh*** | 454 | 363 | 201 | 367 | 46 | 819 | 44 | 210 | 70 | 196 |
|  | ***pdhC*** | 459 | 340 | 199 | 342 | 43 | 818 | 52 | 215 | 81 | 183 |
|  | ***pgm*** | 494 | 347 | 226 | 362 | 54 | 809 | 54 | 212 | 67 | 179 |
| **NG-MAST** | ***por*** | 509 | 344 | 268 | 389 | 52 | 843 | 55 | 242 | 94 | 221 |
|  | ***tbpb*** | 521 | 363 | 157 | 385 | 65 | 801 | 50 | 203 | 69 | 160 |
| **NG-STAR** | **23S rRNA1** | 551 | 388 | 244 | 346 | 66 | 1138 | 52 | 242 | 90 | 245 |
|  | ***gyrA*** | 473 | 303 | 209 | 360 | 42 | 838 | 45 | 184 | 82 | 226 |
|  | ***mtrR*** | 494 | 338 | 229 | 364 | 44 | 790 | 38 | 229 | 111 | 218 |
|  | ***parC*** | 476 | 326 | 247 | 308 | 44 | 777 | 53 | 187 | 73 | 203 |
|  | ***penA*** | 582 | 384 | 209 | 273 | 56 | 867 | 42 | 217 | 92 | 125 |
|  | ***ponA*** | 536 | 337 | 225 | 373 | 47 | 829 | 56 | 209 | 98 | 208 |
|  | ***porB* (full_gene)** | 509 | 345 | 262 | 360 | 50 | 904 | 55 | 241 | 96 | 226 |

1. **Mutations resulting in AMR**

Multiple mutations have been reported in many genes conferring resistance to different classes of antibiotics in *N. gonorrhoeae*. A brief overview of the mutations in the genes and their possible modes of action is provided in this section.

Quinolones (eg.Ciprofloxacin) affect bacterial DNA replication by interacting with proteins like DNA gyrase (GyrA) and topoisomerase IV (ParC). Mutations in these proteins in the quinolone resistance determining region (QRDR), which alter the quinolone binding site, have been shown to cause resistance^10^. The β-Lactam antibiotics, penicillins and cephalosporins, inhibit bacterial cell wall synthesis by binding to transpeptidase enzymes (penicillin-binding proteins or PBPs). TEM-1 gene-containing plasmids encode a TEM-1-type β-lactamase which hydrolyzes the β-lactam ring and renders the penicillin inactive^11^. Mutations in genes encoding penicillin-binding proteins (*penA*, *ponA1*, *penC*/*pilQ*), *mtrR* (repressor of MtrCDE efflux pump) and the porin, *porB1b* result in resistance to β-lactam antimicrobials^10^. Mutations in *mtrR* promoter result in overexpression of MtrCDE efflux pump, causing multi-drug resistance; while porin mutations cause a slightly lower influx of antibiotics in the cell^12^.

Tetracyclines inhibit protein synthesis by binding to the 30S ribosomal subunit. Plasmid-encoded *tet*(M) resembles elongation factor G (EF-G) and binds to the ribosomes causing the release of the tetracycline molecule. Mutations in ribosomal binding protein S10, encoded by *rpsJ* (V57M) results in tetracycline resistance, in strains with mutations in *penB* and *mtrR*^10^. Spectinomycin inhibits translation by binding to the 30S ribosomal subunit, specifically to the 16S rRNA and 30S ribosomal protein S5p (*rpsE*). Mutations in these two genes result in resistance to spectinomycin. Macrolides inhibit protein synthesis by binding to the 50S ribosomal subunit, specifically 23S rRNA. Macrolide resistance results from mutations in 23S rRNA or by the acquisition of conjugative transposon encoded proteins which cause either modification of 23S rRNA by rRNA methylase (*erm* genes) blocking macrolide interaction or overexpression of efflux pump system (*mef* pump)^13^.

Plasmid encoded aminoglycoside N-acetyltransferases (AACs) can result in result in aminoglycoside resistance including gentamycin^14^. Gentamycin resistance can also result due to mutations in 16S rRNA and 23S rRNA described above. Mutations in RNA polymerase subunit beta (*rpoB*) and RNA polymerase delta (*rpoD*) can result in resistance to extended spectrum cephalosporin (ESC)^15^. Specific mutations in *rpoB* can also result in rifampicin resistance^16^.

Sulfonamides inhibit the synthesis of folic acid, by targeting the dihydropteroate synthase. Mutations in this antibiotic target, *folP*, increases sulphonamide MIC^17^. Mutations in the *mtrR* DNA-binding domain affects repression resulting in overexpression of the MtrCDE efflux pump system leading to multi-drug resistance. Promoter mutations in the genes coding for efflux pumps NorM and MacAB cause resistance to fluoroquinolones and macrolides, respectively^10^.

**Supplementary Table 5a: List of the AMR profiling identified through literature.** This table contains the experimentally verified mutations in AMR target genes in *N. gonorrhoeae* and the references.

| **Antibiotic class** | **Reference** | **Gene** | **Mutation** | **Residue in the reference genome** | **Remarks** |
| --- | --- | --- | --- | --- | --- |
| **Ciprofloxacin** | Unemo and Shafer^10^, 2012; Kivata et al^18^, 2019 | *gyrA* | S91F/Y | S | High MIC values |
|  | Unemo and Shafer, 2012; Kivata et al, 2019; Harrison et al, 2016^19^ |  | D95N/G/A/Y | D | High MIC values |
| **Ciprofloxacin** | Unemo and Shafer, 2012; Tanaka et al, 2000; Harrison et al, 2016 | *parC* | D86N | D | Strains with intermediate resistance, in combination with other mutations in gyrA and parC |
|  | Zhou et al^20^, 2004; Tanaka et al, 2000; Harrison et al, 2016 |  | S87R/I/N | S | Resistant strains, in combination with gyrA mutations |
|  | Unemo and Shafer, 2012, Tanaka et al, 2000 |  | S88P | S | Resistant strains, in combination with gyrA mutations |
|  | Unemo and Shafer, 2012; Kivata et al, 2019, Tanaka et al 2000 |  | E91K/G/Q | E | Resistant strains, in combination with gyrA mutations |
| **Penicillin and extended spectrum cephalosporins (ESCs)** | Unemo and Shafer, 2012 | *ponA1* (PBP1) | L421P | L | Reduced susceptibility |
| **Tetracycline** | Unemo and Shafer, 2012 | *rpsJ* | V57M | V | Reduced susceptibility |
| **Penicillin, cephalosporins** | Unemo and Shafer, 2012 | *penA* (PBP2) | A311V | A | High MIC values for ceftriaxone and cefixime |
|  | Unemo and Shafer, 2012 |  | I312M | I | High MIC values cefixime |
|  | Unemo and Shafer, 2012 |  | V316T/P | V | High MIC values ceftriaxone and cefixime |
|  | Unemo and Shafer, 2012 |  | D345 insertion | D | High MIC values for penicillin |
|  | Unemo and Shafer, 2012 |  | T483S | T | High MIC values ceftriaxone and cefixime |
|  | Unemo and Shafer, 2012 |  | G545S | G | High MIC values cefixime |
| **Tetracycline, penicillin, cephalosporins** | Unemo and Shafer, 2012; Harrison et al, 2016 | *porB* | G120D/K/N/R | G | Strains with moderate resistance, high MIC in combination with A121 mutation |
|  |  |  | A121D/G/N/S | A | Strains with moderate resistance MIC, high MIC in combination with G120 mutation |
| **Sulfonamide** |  | *folP* | R228S | R | Strains with high MICs |
| **Macrolide** | Unemo and Shafer, 2012, Ma et al 2020 | 23S rRNA | A2059G | - | High MIC, Reduced affinity for macrolides |
|  |  |  | C2611T |  | High MIC, Reduced affinity for macrolides |
| **Macrolide** | Unemo and Shafer, 2012, Ma et al 2020 | *macAB* -35 operon | G → T substitution in −10 promoter region, increases transcription, TAGAAT to TATAAT | T | High expression of *macAB*, macrolide resistance |
| **Penicillin, tetracycline, macrolides, cephalosporins** | Unemo and Shafer, 2012 | *mtrR* and *mtrCDE* promoter | C → T at mtr120 | - | High expression of *mtrCDE* |
|  | Unemo and Shafer, 2012 |  | 153-bp Correia element insertion between the mtrR/mtrC promoter and the mtrC gene | - | High expression of *mtrCDE* |
|  | Unemo and Shafer, 2012 |  | del of A in 13 bp inv repeat | - | High expression of *mtrCDE* |
|  | Loughlin et al, 2018; Warner et al, 2008 |  | T → G, -35 hexamer of mtrCDE  TTTTAT → TTGTAT |  | High expression of *mtrCDE* |
|  | Kivata et al, 2019; Warner et al, 2008^22^ | *mtrR* | A39T | A | Seen in intermediate and fully resistant strains |
|  | Unemo and Shafer, 2012 |  | G45D | G | Seen in intermediate and fully resistant strains |
|  | Warner et al, 2008 |  | E202G | E | High MIC values |
|  | Loughlin et al, 2018; Wadsworth et al | *mtrD* | S821A | - | High MIC values |
|  | Loughlin et al, 2018; Wadsworth et al |  | K823E/D | - | High MIC values |
| **Spectinomycin** | Unemo and Shafer, 2012 | 16S rRNA | C1192U(G1192A) | - | High MIC values |
| **Spectinomycin** | Unemo and Shafer, 2012, Harrison et al 2016 | S5p/rpsE | V27Δ | V | High MIC values |
|  | Unemo and Shafer, 2012, Harrison et al 2016 |  | K28E | K | High MIC values |
| **Azithromycin** | Ma et al, 2020 | L4p/*rplD* | G70D/S/A/R/**Y** | G | High MIC values |
| **ESCs** | Palace et al, 2020 | *rpoB* | R201H |  | High MIC values |
| **ESCs** | Palace et al, 2020 | *rpoD* | Δ92-95 | - | High MIC values |
|  |  |  | E98K | E | High MIC values |
| **Penicillin** | Cehovin et al, 2018 | beta-lactamase containing plasmids |  |  | Plasmid encoding the beta lactamase protein |
| **Tetracycline** | Cehovin et al, 2018 | tet-M containing plasmids |  |  | Plasmid encoding the tetracycline resistance protein Tet(M) |
| **Aminoglycosides** | Garneau-Tsodikova et al, 2016 | AAC containing plasmids | plnc |  | Plasmid encoding aminoglycoside modifying enzymes like aminoglycoside N-acetyltransferases |

**Supplementary Table 5b: Sequencing depth of the regions with mutations implicated in AMR.** Sequencing depth for the gene loci, *rpsJ* and *folP*, with AMR mutations listed in Table 3 of the main manuscript. The depth of other gene loci with mutations listed in Table 3 have already been covered in Supplementary Table 4d.

| **Strain id** | | | | | | | | | | |
| --- | --- | --- | --- | --- | --- | --- | --- | --- | --- | --- |
| **Gene** | **3** | **12** | **18** | **57** | **61** | **100A** | **240** | **274** | **285** | **298** |
| ***rpsJ*** | 533 | 330 | 255 | 1341 | 67 | 866 | 73 | 257 | 118 | 241 |
| ***folP*** | 489 | 370 | 196 | 1247 | 34 | 843 | 50 | 215 | 75 | 168 |
| ***pilQ*** | 541 | 395 | 262 | 1388 | 52 | 909 | 54 | 232 | 111 | 224 |
| ***rpoB*** | 568 | 368 | 236 | 1425 | 67 | 980 | 60 | 224 | 99 | 216 |

**Supplementary Table 5c: A comparison of results from AMR profiling using the databases Pathogenwatch, ARIBA and CARD database and this study.** The AMR mutations predicted by the databases and our dataset is given below. R Resistant, I Intermediate, - Susceptible. Out of the predicted ciprofloxacin-resistant strains, strains 12 and 57 are resistant and strains 298 and 100A intermediate resistance. Among the mutations observed in the strains sequenced in the study, the accessory ParC mutations for high ciprofloxacin resistance in strains with gyrA mutations, few *penA* mutations, *mtrR* and *rplD* mutations and *rpoB* mutations have been missed by Pathogenwatch^30^. ARIBA^31^ dataset has missed the *parC* E91G mutation and a few mutations in *penA*, *mtrR* and *rplD*. CARD database^32^ has missed mutations in *rpoB*, *gyrA* (D95A), *penA* insertion 365D, *rpoB* and *porB1b* mutations. The entire list of mutations observed in the clinical strains studied here has been provided in Table 3 in the main manuscript.

| **Strain ids.** | | | | | | | | | | | |
| --- | --- | --- | --- | --- | --- | --- | --- | --- | --- | --- | --- |
|  |  | **3** | **12** | **18** | **57** | **61** | **100A** | **240** | **274** | **285** | **298** |
| **Antibiotic** | **Dataset** |  |  |  |  |  |  |  |  |  |  |
| Ciprofloxacin | Pathogenwatch |  | gyrA S91F, D95A | gyrA S91F, D95A | gyrA S91F, D95A |  | gyrA S91F, D95A | gyrA S91F |  | gyrA S91F |  |
|  | CARD |  | gyrA S91F  parC E91G | gyrA S91F | gyrA S91F |  | gyrA S91F | gyrA S91F  parC E91G | parC E91G | gyrA S91F  parC E91G |  |
|  | ARIBA |  | gyrA S91F | gyrA S91F | gyrA S91F |  | gyrA S91F | gyrA S91F |  | gyrA S91F |  |
|  | Our method |  | gyrA S91F, D95A  parC E91G | gyrA S91F, D95A | gyrA S91F, D95A |  | gyrA S91F, D95A | gyrA S91F  parC E91G | parC E91G | gyrA S91F  parC E91G |  |
| Penicillin | Pathogenwatch | penA ins346D  ponAL421P | penA ins346D | penA ins346D  ponAL421P  porB1bA121D | penA ins346D  ponAL421P | penA ins346D | penA ins346D  ponAL421P | penA ins346D  ponAL421P | penA ins346D | penA ins346D  ponAL421P | penA ins346D |
|  | CARD | PenA F504L, A501V, A516G  ponAL421P | PenAF504L, A501V, A516G | PenAF504L, A501V, A516G  ponAL421P | PenAF504L, A501V, A516G  ponAL421P | PenAF504L, A501V, A516G | PenAF504L, A501V, A516G  ponAL421P | PenAF504L, A501V, A516G, P551L  ponAL421P | PenAF504L, A501V, A516G  ponAL421P | PenAF504L, A501V, A516G, P551L  ponAL421P | PenAF504L, A501V, A516G |
|  | ARIBA | PenA A501V  ponAL421P | PenAA501V | PenA A501V, ponA L421P, porB1bA121D | PenA A501V  ponAL421P | PenA A501V | PenA A501V  ponAL421P | PenA A501V  ponAL421P | PenA A501V  ponAL421P | PenA A501V  PonA L421P | PenA A501V |
|  | Our method | penA ins346D, F504L, A501V, A516G  ponAL421P | penA ins346D, F504L, A501V, A516G | penA ins346D, F504L, A501V, A516G  ponAL421P  porB1bA121D | penA ins346D, F504L, A501V, A516G  ponAL421P | penA ins346D, F504L, A501V, A516G | penA ins346D, F504L, A501V, A516G  ponAL421P | penA ins346D, F504L, A501V, A516G, P551L  ponAL421P | penA ins346D, F504L, A501V, A516G | penA ins346D, F504L, A501V, A516G, P551L  ponAL421P | penA ins346D, F504L, A501V, A516G |
| Sulfonamide | Pathogenwatch | folP R228S | folP R228S | folP R228S | folP R228S | folP R228S | folP R228S | folP R228S | folP R228S | folP R228S | folP R228S |
|  | CARD |  |  |  |  |  |  |  |  |  |  |
|  | ARIBA | folP R228S | folP R228S | folP R228S | folP R228S | folP R228S | folP R228S | folP R228S | folP R228S | folP R228S | folP R228S |
|  | Our method | folP R228S | folP R228S | folP R228S | folP R228S | folP R228S | folP R228S | folP R228S | folP R228S | folP R228S | folP R228S |
| Tetracycline | Pathogenwatch | rpsJV57M | rpsJV57M | rpsJ V57M | rpsJV57M | rpsJV57M | rpsJV57M | rpsJV57M | rpsJV57M | rpsJV57M | rpsJV57M |
|  | CARD | rpsJV57M | rpsJV57M | rpsJV57M | rpsJV57M | rpsJV57M | rpsJV57M | rpsJV57M | rpsJV57M | rpsJV57M | rpsJV57M |
|  | ARIBA | rpsJV57M | rpsJV57M | rpsJ V57M | rpsJV57M | rpsJV57M | rpsJV57M | rpsJV57M | rpsJV57M | rpsJV57M | rpsJV57M |
|  | Our method | rpsJV57M | rpsJV57M | rpsJV57M | rpsJV57M | rpsJV57M | rpsJV57M | rpsJV57M | rpsJV57M | rpsJV57M | rpsJV57M |
| Macrolide | Pathogenwatch |  |  |  |  |  |  |  |  |  |  |
|  | CARD | mtrR A39T  rplD G70D | mtrR A39T | mtrR A39T | mtrR A39T | mtrR A39T | mtrR A39T |  |  |  |  |
|  | ARIBA |  |  |  |  |  |  |  |  |  |  |
|  | Our method | mtrR A39T  rplD G70D | mtrR A39T | mtrR A39T | mtrR A39T | mtrR A39T | mtrR A39T |  |  |  |  |

1. **Basis of drug resistance derived from literature**

We identified mutations in eleven proteins associated with antibiotic resistance - GyrA, ParC, PBP1 (*ponA1*), RpsJ, PBP2 (*penA*), PorB1b, FolP, RplD, RpoB, PilQ and MtrR. The mechanistic basis for resistance has been predicted for RpsJ (V57M) (tetracycline resistance), RplD (azithromycin resistance), mtrR (macrolide resistance), pilQ (penicillin and ESC resistance) and PBP2 mutations. The mechanistic basis for resistance has already been predicted for RpsJ (V57M) (tetracycline resistance), RplD (azithromycin resistance), MtrR (macrolide resistance) and PBP2 mutations.

Ribosomal protein S10 (*rpsJ*)- V57M mutation is associated with resistance to tetracycline. From studies on the structure of the 30S ribosomal subunit from *Thermus thermophilus* (PDB id: 1HNW)^33^, this residue was seen to be close to the tetracycline-binding site in RpsJ. The presence of hydrophobic side chains at this site is thought to alter the structure and reduce the binding affinity of tetracycline^34^.

*penA*- ^35^. The D345a residue is involved in a hydrogen bond network with catalytic serine residue, which may prevent the acylation of R1 subunit of penicillin, resulting in resistance. The mutation F504L occurs in the β3–β4 loop. This loop is thought to be important for the flexibility and resistance to beta-lactams, as inferred from studies in *Streptococcus pneumoniae*^36,37^.

*mtrR*- The MtrR protein from *N. gonorrhoeae* has been crystallised (PDB id: 6OF0)^38^. The mutations A39T and H105 are observed in the α2 helix which is a part of the DNA binding domain and α6, respectively, which is closer to the DNA binding region, in the wild-type protein structure. A39T mutation is thought to decrease the affinity of MtrR for *mtrCDE* promoter region, leading to resistance^38^.

*pilQ* mutations are thought to interfere with pilus assembly and in combination with mutations in other genes such as *penA* and *mtrR* results in resistance to penicillins and extended spectrum cephalosporins.

1. **Investigation of the structural basis for antibiotic resistance mutations**

Mutation is one of the most common mechanism of antibiotic resistance hence we attempted to study the effect of mutations on the interactions of the drug with the protein targets. We also investigated the basis of drug resistance for the protein mutations in GyrA, ParC, FolP, PonA1 and PorB1b and the novel mutations identified in this study through homology modelling using templates with co-crystallized antibiotic structures.

**Supplementary Table 6: Template details of homology modelling for the proteins with mutations involved in AMR observed in the sequenced strains.**

| **Protein and gene name** | **PDB id** | **Resolution** | **PDB details** | **Reference organism** | **% identity** |
| --- | --- | --- | --- | --- | --- |
| **DNA gyrase A (*gyrA*)** | 2XCT | 3.35Å | Gyrase (GyrA-GyrB fusion truncate) with moxifloxacin and DNA | *Staphylococcus aureus* | 47.23 |
| **DNA Topoisomerase IV subunit ParC (*parC*)** | 2XKK | 3.25Å | Topo IV (ParE-ParC fusion truncate) with moxifloxacin and DNA | *Acinetobacter baumannii* | 51.95 |
| **Penicillin-binding protein (*ponA1*)** | 3UDI | 2.60Å | PBP1a in complex with penicillin G | *A. baumannii* | 40.54 |
| **Porin (*porB1b*)** | 4GCP | 1.98Å | Outer membrane protein F (OmpF) porin with Ampicillin | *E. coli* | 22 |
| **Dihydropteroate synthase (*folP*)** | 3TZF | 2.10Å | Dihydropteroate Synthase with Sulfonamide Drug Complex | *Yersinia pestis* | 47.17 |

*gyrA*- *S. aureus* gyrase A and gyrase B dimer complexed with ciprofloxacin and DNA (PDB:2XCT)^60^ was taken as the template for modelling GyrA proteins from reference strain and strain 12 (S91F and D95G). The substrates DNA and ciprofloxacin were retained in the modelled structure (Supplementary Figure 3a). The mutations S91F/Y and D95N/G/A have been reported to confer ciprofloxacin resistance. In the wild-type structure, the residue S91 formed polar contacts with the drug ciprofloxacin (Supplementary Figure 3b). In the mutant, S91F, however, this contact was abolished (Supplementary Figure 3c). We hypothesize that the mutation might result in the weakening of drug-protein interaction, thereby resulting in drug resistance.


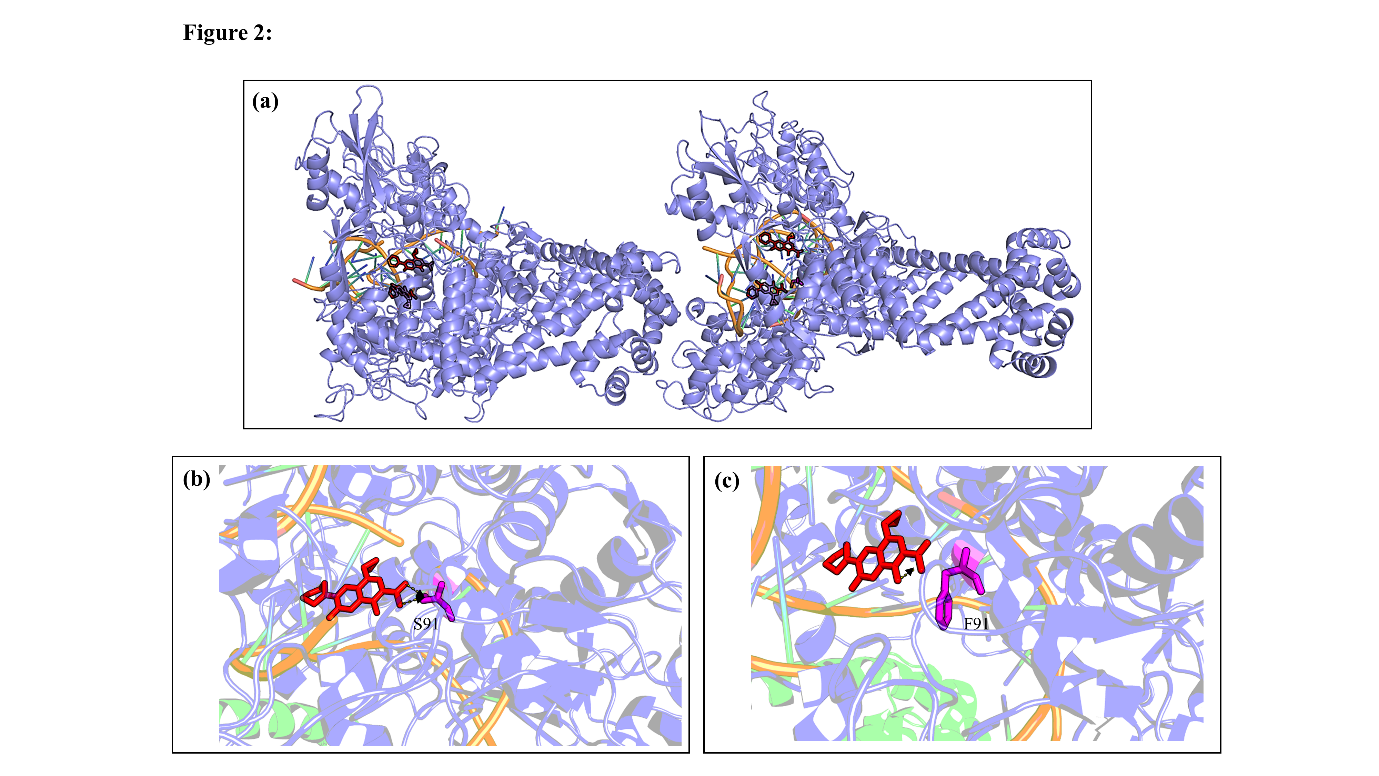


**Supplementary Figure 3: The structural basis of quinolone resistance mediated by GyrA**

The modelled structure of the GyrA-GyrB complex with ciprofloxacin and DNA (a). A close-up of the antibiotic-binding region showing ciprofloxacin (red), S91 (magenta) in wild-type structure (b) and S91F mutant (magenta) (c). The polar contacts for the antibiotic are shown as yellow dashes.

*parC*- ParC and ParE (DNA topoisomerase IV subunits A and B, respectively) are homologues of GyrA and GyrB^61, 62^. *A. baumannii* topoisomerase IV (ParE-ParC fusion truncate) co-crystallized with moxifloxacin and DNA (PDB id: 2XKK)^63^ was used as the template to model sequences from the reference strain and strain 12 (E91G). We observe that E91G mutation occurs close to S88, and these two residues are involved in a hydrogen bond network with the antibiotic, moxifloxacin (Supplementary Figures 4a-c). E91 is also within 5Å of an Mg^2+^ ion which interacts with the antibiotic. In the case of the mutant, the hydrogen bonding network with the antibiotic is lost and the antibiotic interacts only with Mg^2+^. Thus, the interaction with moxifloxacin and other quinolones may be weakened in the case of the mutation. The novel mutation V68A occurs in the QRDR region of ParC, we were not able to observe the effect of this mutation in the model as the template does not have the corresponding residue.

**
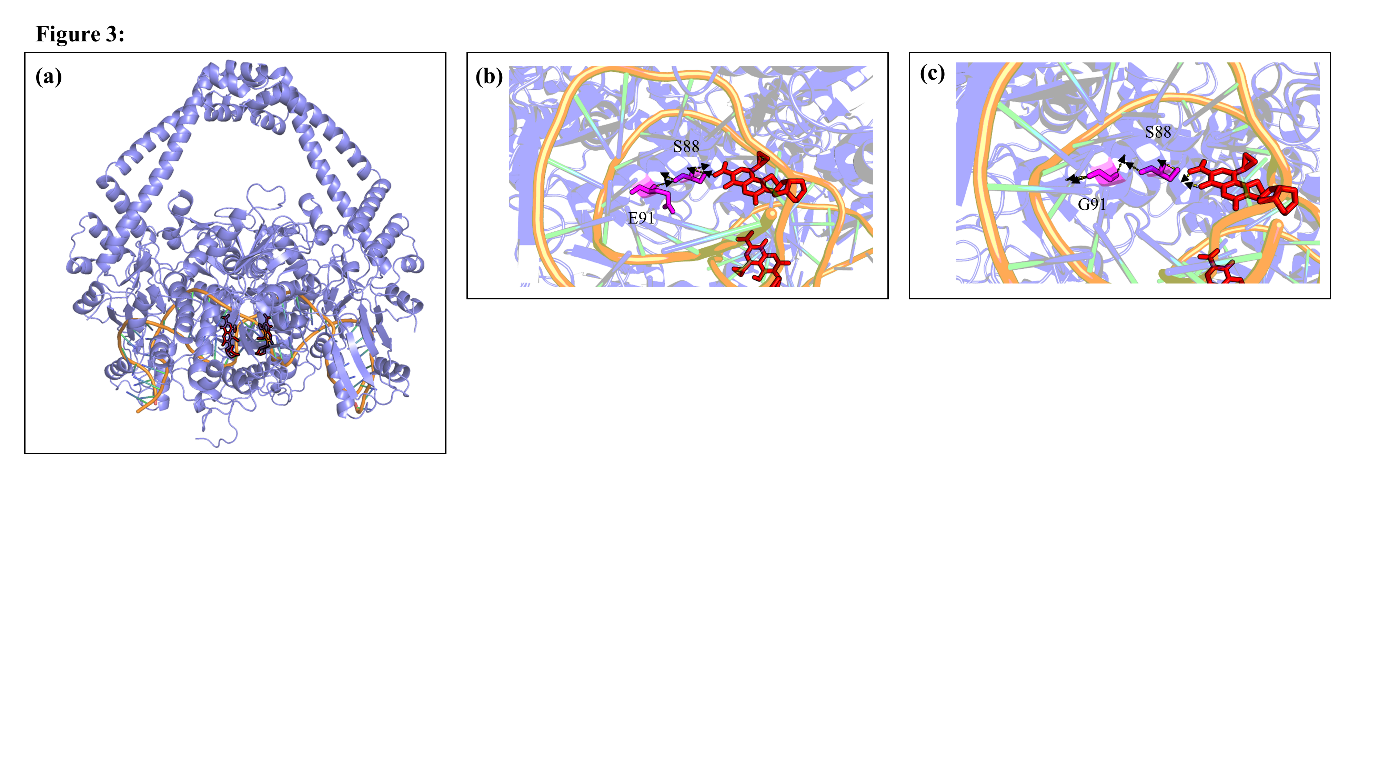
**

**Supplementary Figure 4: The mechanistic basis of quinolone resistance mediated by ParC.** The modelled structure of the ParC-ParE complex with moxifloxacin and DNA (a). A close-up of the antibiotic-binding region showing moxifloxacin (red), S88 (magenta) and E91 in wild-type structure (b) and E91G mutant (magenta) (c). The polar contacts for the antibiotic are shown as yellow dashes.

*folP-* FolP from *Yersinia pestis* crystallized with the substrate, 6-hydroxymethylpterin diphosphate and the sulfa drug, sulfamethoxazole (PDB id: 3TZF) was used as a template^57^ for modelling the wild-type and mutant FolP from the strain 3 (R228S mutation). Both R228 and S228 were seen to interact with the sulfa drug, the R228S mutation may interfere with the binding of the protein to the sulfa drug (Supplementary Figures 5a-c). We have identified a novel mutation, E79 insertion in folP in all the strains sequenced in the study, upon modelling this mutation did not fetch any detectable changes with respect to interaction with substrate or the antibiotic. Broader sequencing of more isolates from Kenya can help us understand the prevalence of the isolates and experimentally validate if there is any effect on sulfonamide resistance.

**
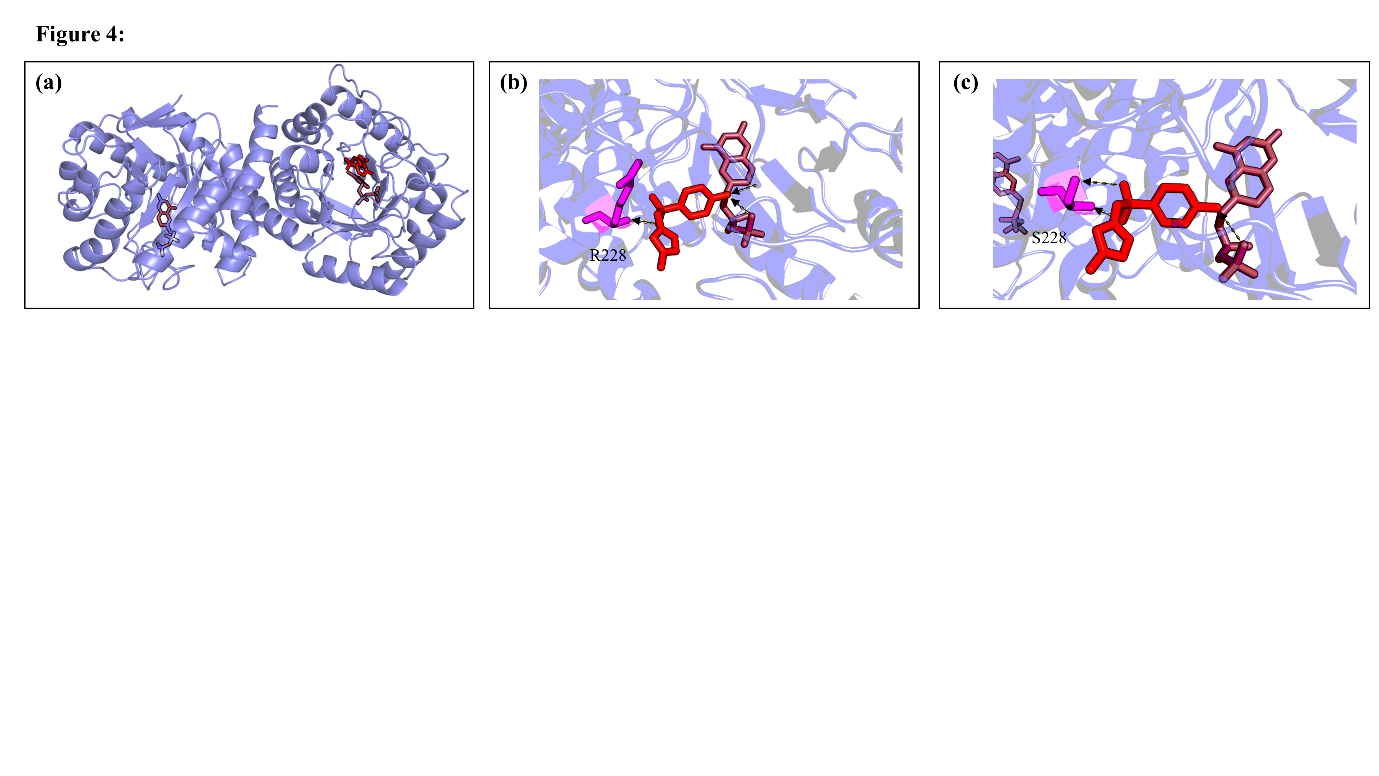
**

**Supplementary Figure 5: The mechanistic basis of quinolone resistance mediated by FolP**

The modelled structure of the FolP complex with sulfamethoxazole and substrate, 6-hydroxymethylpterin diphosphate (a). A close-up of the antibiotic-binding region showing sulfamethoxazole (red), the substrate (maroon) and R228 (magenta) in wild-type structure (b) and R228S mutant (magenta) (c). The polar contacts for the antibiotic are shown as yellow dashes.

*porB1b*- Structure of outer membrane porin F from *E. coli* in complex with ampicillin (PDB id: 4GCP) was used as the template for homology modelling of PorB1b protein from reference strain (G120, A121, N122) and our sequenced strain 57 (D120, G121 and D122). Gly120 and Asp121 and Asn122 mutations confer intermediate resistance to penicillin and tetracycline and are located in the loop3 region which forms the pore constriction zone^39^. The mutated residues are shown in Supplementary Figures 6a-c. These side chains could be lining a substrate translocation pathway, consistent with observations that mutations decrease antibiotic permeability^10^.


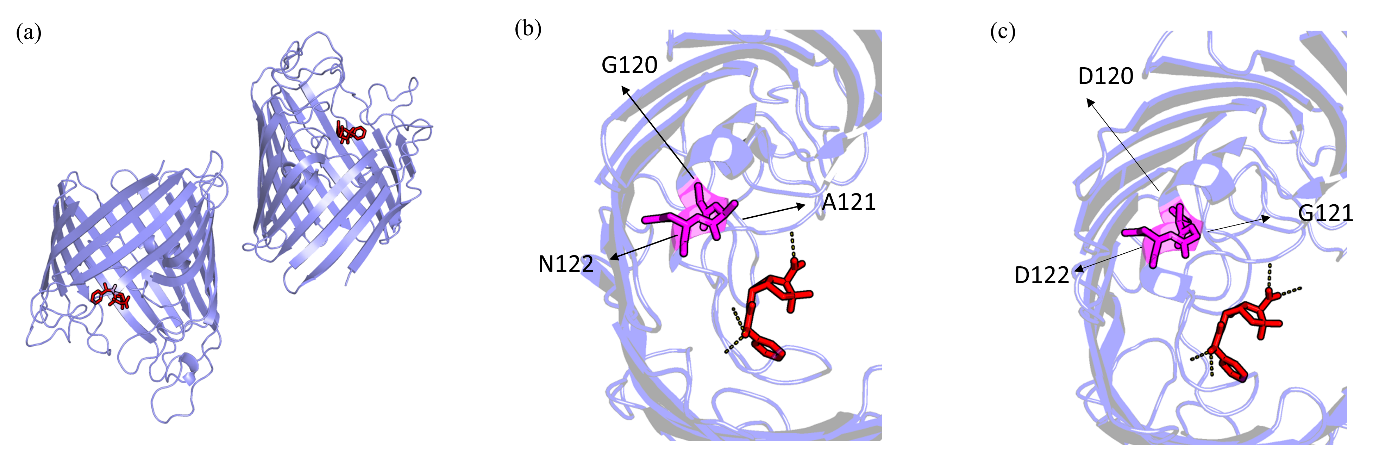


**Supplementary Figure 6:** The modelled structure of the porB1b complex with ampicillin (a). A close-up of the antibiotic-binding pocket showing ampicillin (red) and the residues G120, A121, N122 in reference strain structure (b) and in the triple mutant D120, G121 and D122 (magenta) (c). The polar contacts for the antibiotic are shown as yellow dashes.

*ponA1* has only one reported mutation in *N. gonorrhoeae*, L421P, which occurs about 40 residues to the upstream of catalytic residue S460 and confers resistance to penicillin and extended-spectrum cephalosporins^37^. We modelled the structure of wild-type and mutant *ponA1* from strain 3 using the template, *Acinetobacter baumannii* PBP1a in complex with penicillin G (PDB id: 3UDI)^40^. The homology model revealed that the mutation occurs in the C-terminal end of the ODD (outer-membrane PBP1A docking domain), which acts as a linker between the transpeptidase domain and glycosyltransferase domain (Supplementary Figures 7a-c)^40^. The domain has been suggested to serve as a structural intermediary between the two domains^36^ and in the formation of a hydrophobic patch to selectively regulate substrate entry into the catalytic pocket^41^, based on studies in other bacteria.


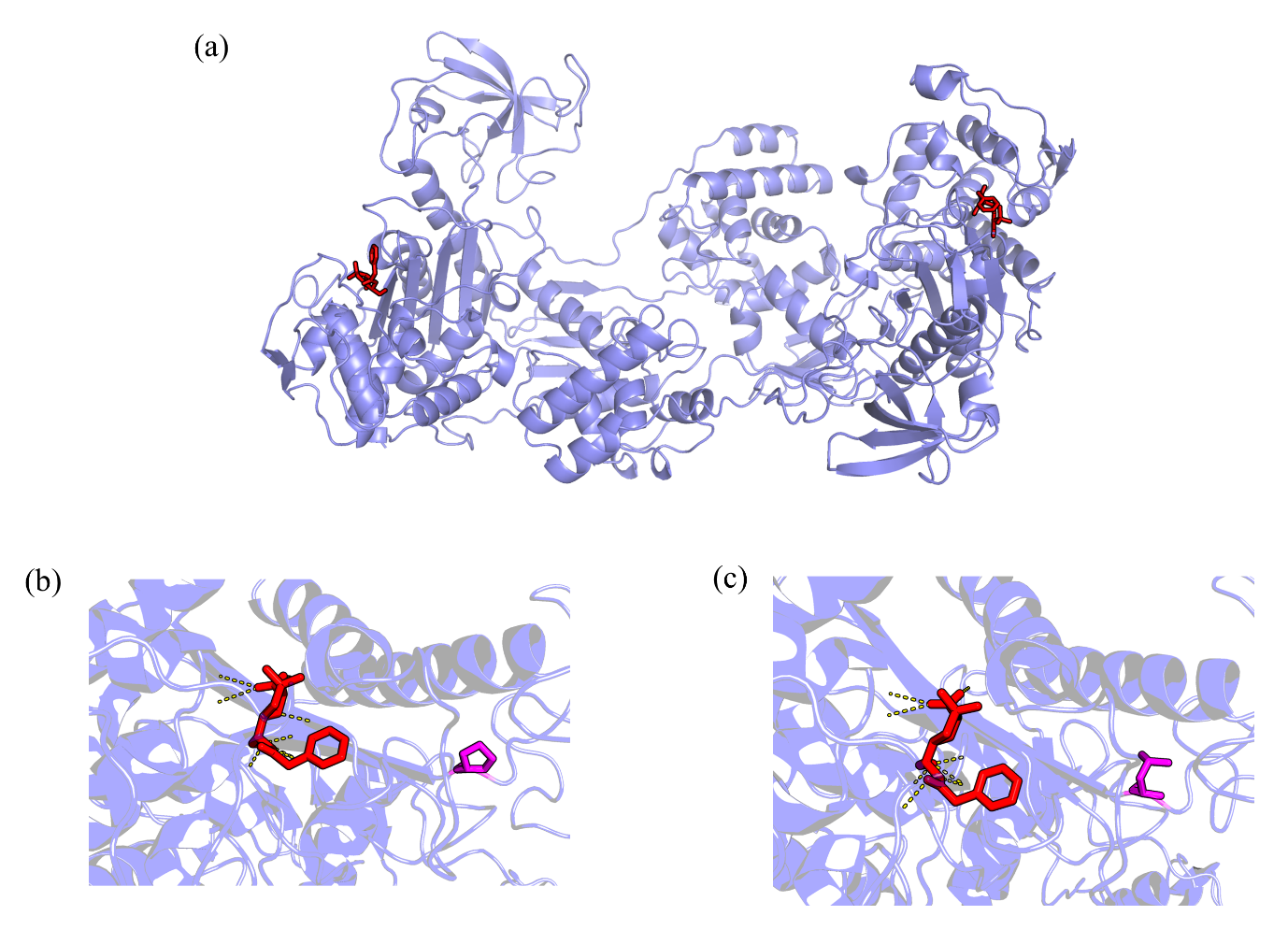


**Supplementary Figure 7:** The modelled structure of the *ponA1* complex with penicillin (a). A close-up of the antibiotic-binding pocket showing penicillin G (red), L421 (magenta) in wild-type structure (b) and L421P mutant (magenta) (c). The polar contacts for the antibiotic are shown as yellow dashes.

*rpoB*- Studies in *Mycobacterium tuberculosis*, showed the structural basis of drug resistance in the case of several *rpoB* mutations. The H526Y (H522Y in *N. gonorrhoeae*- Supplementary Figure 8) mutation causes stearic hindrance in the rifampicin binding pocket preventing the binding.


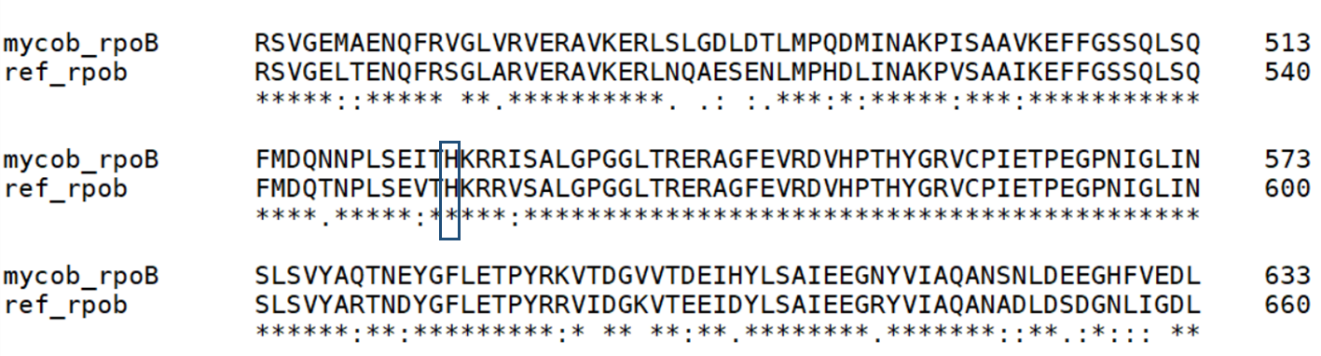


**Supplementary Figure 8: Pairwise sequence alignment of the *rpoB* sequences from *M. tuberculosis* and *N. gonorrhoeae*.** The alignment was generated using Clustal Omega and the RpoB sequence from PDB id 5UAC. H526 (H222 in *N. gonorrhoeae*) residue has been indicated in the alignment. Mycob_rpoB- mycobacteria RpoB sequence, ref_rpob- gonococci RpoB sequence.

Ribosomal protein L4p (*rplD)*- G70 residue is predicted to make contacts with azithromycin bound to the 50S ribosome from studies in *Thermus thermophilus*^42^. The mutations G70D/A/S/R have been reported, we identified a novel mutation at the position, G70Y. We hypothesize that the substitutions of amino acids with side chains at this position affect the binding of azithromycin in the binding pocket due to steric hindrance.

1. **Phylogenetic analysis with strains across the world**

**Supplementary Table 7: Details for strains used in phylogeny analysis.** The analysis was carried out using the genome comparator module of BIGSdb, and details like country and antibiotic resistance were derived from PubMLST using a keyword search for strain accession identifiers (denoted in the table as id). Colour code: Dark yellow with red text- Resistant, Cream with green text- Intermediate. ^*^-*N. meningitidis* strain, used as outgroup. ^**^- *N. gonorrhoeae* reference strain.

| **id** | **isolate** | **country** | **penicillin** | **ceftriaxone** | **ciprofloxacin** | **tetracycline** | **cefixime** | **azithromycin** | **ST (MLST)** | **Cluster in the phylogeny** |
| --- | --- | --- | --- | --- | --- | --- | --- | --- | --- | --- |
| 12672^*^ | 053442 | China |  |  |  |  |  |  | 1899 |  |
| 2855^**^ | FA1090 | USA |  |  |  |  |  |  | 4821 | VI |
| 27394 | GCGS0235 |  | 2 | 0.06 | 16 | 4 | 0.25 | 0.25 | 1901 | III |
| 27395 | GCGS0076 |  | 1 | 0.008 | 8 | 2 | 0.015 | 0.25 | 1901 | III |
| 27396 | GCGS0149 |  | 1 | 0.03 | 0.015 | 1 | 0.25 | 0.5 | 1580 | III |
| 27397 | GCGS0229 |  | 2 | 0.06 | 16 | 2 | 0.25 | 0.5 | 1901 | III |
| 27398 | GCGS0139 |  | 4 | 0.125 | 32 | 4 | 0.25 | 1 |  | III |
| 27399 | GCGS0185 |  | 2 | 0.06 | 16 | 2 | 0.25 | 0.25 |  | III |
| 27400 | GCGS0081 |  | 2 | 0.06 | 16 | 1 | 0.25 | 0.125 |  | III |
| 27401 | GCGS0077 |  | 4 | 0.06 | 16 | 2 | 0.25 | 1 |  | III |
| 27402 | GCGS0201 |  | 1 | 0.063 | 0.004 | 1 | 0.25 | 0.5 | 1580 | III |
| 27403 | GCGS0213 |  | 1 | 0.063 | 0.004 | 0.5 | 0.5 | 0.5 |  | III |
| 31461 | WHO_O | Canada | 32 | 0.032 | 0.008 |  |  |  | 1902 | VIII |
| 31505 | 318/-10 | France | 1 | 2 | 32 | 2 | 3 | 0.38 | 1901 | III |
| 31562 | WHO_F | Canada | 0.032 | 0.002 | 0.004 |  |  |  | 10934 | IV |
| 31566 | WHO_P | USA | 0.25 | 0.004 | 0.004 |  |  |  | 8127 | III |
| 31586 | 989000380 | Russia | 0.75 | 0.004 | 0.002 |  |  |  | 11188 | III |
| 31587 | 989000257 |  | 0.125 | 0.002 | 0.003 |  |  |  | 9903 | III |
| 31588 | 989000326 |  | 0.016 | 0.002 | 0.002 |  |  |  | 1892 | IV |
| 31596 | 05_590_246 |  | 0.75 | 0.016 | 6 | 1 | 0.016 | 0.125 | 1901 | III |
| 31597 | 05_590_269 |  | 0.38 | 0.008 | 8 | 1 | 0.016 | 0.125 |  | III |
| 31598 | 05_590_290 |  | 0.125 | 0.003 | 0.006 | 1 | 0.016 | 0.38 | 6810 | V |
| 31663 | WHO M | Australia | 8 | 0.012 | 2 | 1 | 0.016 | 0.25 | 7367 | III |
| 31664 | WHO L |  | 2 | 0.125 | 32 | 4 | 0.25 | 0.5 | 1590 | VI |
| 31665 | WHO J |  | 32 | 0.023 | 24 | 3 | 0.016 | 0.38 | 1599 | III |
| 31666 | WHO I |  | 3 | 0.032 | 0.75 | 3 | 0.032 | 0.25 | 1890 | VI |
| 31695 | WHO_K | Japan | 2 | 0.064 | 32 | 2 | 0.5 | 0.25 | 7363 | III |
| 31758 | WHO_G | Australia | 0.5 | 0.008 | 0.125 | 32 | 0.016 | 0.25 | 1903 | III |
| 31761 | 05_590_342 | Russia | 0.064 | 0.032 | 0.75 | 1 | 0.023 | 1 | 1902 | VIII |
| 31764 | WHO_N | Australia | 8 | 0.004 | 4 | 16 | 0.016 | 0.125 | 1583 | III |
| 31768 | 05_590_264 | Russia | 0.064 | 0.094 | 0.75 | 1 | 0.023 | 1 | 8160 | III |
| 31799 | 05_590_339 |  | 0.12 | 0.002 | 0.003 | 1 | 0.016 | 0.094 | 6810 | V |
| 31805 | 05_590_249 |  | 2 | 0.032 | 0.016 | 3 | 0.023 | 0.5 | 1905 | IV |
| 31931 | WHO M | Unknown |  |  |  |  |  |  | 7367 | III |
| 31932 | WHO L |  |  |  |  |  |  |  | 1590 | VI |
| 31933 | WHO J |  |  |  |  |  |  |  | 1599 | III |
| 31934 | WHO I |  |  |  |  |  |  |  | 1890 | VI |
| 36238 | 51212 | Kenya |  |  |  |  |  |  | 11364 | II |
| 36240 | 52262 |  |  |  |  |  |  |  | 1903 | VIII |
| 36243 | 57547 |  |  |  |  |  |  |  | 1893 | II |
| 36244 | 56873 |  |  |  |  |  |  |  |  | II |
| 36245 | 44568 |  |  |  |  |  |  |  |  | VIII |
| 36246 | 61467 |  |  |  |  |  |  |  | 1893 | II |
| 36247 | 62017 |  |  |  |  |  |  |  | 11366 | I |
| 36248 | 43305 |  |  |  |  |  |  |  | 1893 | II |
| 36249 | 51451 |  |  |  |  |  |  |  | 1893 | II |
| 36250 | 58767 |  |  |  |  |  |  |  | 11365 | VII |
| 36251 | 63179 |  |  |  |  | 0.25 |  | 0.032 | 11367 | III |
| 36253 | 61450 |  |  |  |  |  |  |  | 11364 | II |
| 36255 | 45453 |  |  |  |  | 12 |  | 0.032 | 1893 | II |
| 36256 | 45676 |  |  |  |  |  |  |  | 1893 | II |
| 36257 | 51960 |  |  |  |  |  |  |  | 1591 | VI |
| 36259 | 61580 |  |  |  |  |  |  |  | 1893 | II |
| 36261 | 48871 |  |  |  |  |  |  |  |  | II |
| 36263 | 48501 |  |  |  |  |  |  |  |  | II |
| 36266 | 60096 |  |  |  |  |  |  |  | 11369 | II |
| 36267 | 55496 |  |  |  |  |  |  |  | 1903 | VIII |
| 36268 | T060 |  |  |  |  |  |  |  | 1599 | IV |
| 36269 | 52263 |  |  |  |  |  |  |  | 11370 | II |
| 36270 | 59688 |  |  |  |  |  |  |  | 8133 | VII |
| 36271 | 50659 |  | 64 |  |  | 12 |  | 0.094 | 6957 | I |
| 36273 | 55798 |  |  |  |  |  |  |  | 11370 | II |
| 36274 | 63518 |  |  |  |  |  |  |  | 11366 | I |
| 36275 | 51468 |  | 0.19 |  |  | 12 |  | 0.19 | 11369 | II |
| 36276 | 51645 |  |  |  |  |  |  |  | 1903 | VIII |
| 36277 | 59012 |  |  |  |  |  |  |  | 11366 | I |
| 36279 | 60243 |  |  |  |  |  |  |  | 1599 | VIII |
| 36280 | 47956 |  |  |  |  |  |  |  | 1893 | II |
| 36281 | 47686 |  |  |  |  |  |  |  |  | II |
| 36282 | 45029 |  | 4 |  |  | 0.5 |  | 0.047 | 11075 | VI |
| 36283 | 47147 |  |  |  |  |  |  |  | 1893 | II |
| 36284 | 44593 |  |  |  |  |  |  |  |  | II |
| 36285 | 43346 |  |  |  |  |  |  |  | 1903 | VIII |
| 36287 | 43781 |  |  |  |  |  |  |  |  | VIII |
| 36289 | 42974 |  | 12 |  |  | 16 |  | 0.125 |  | VIII |
| 36290 | 47983 |  |  |  |  |  |  |  |  | VIII |
| 36291 | 60755 |  |  |  |  |  |  |  | 1588 | VII |
| 36292 | 60709 |  |  |  |  |  |  |  | 1599 | VIII |
| 36293 | 43000 |  |  |  |  |  |  |  | 1903 | VIII |
| 36294 | 43038 |  |  |  |  |  |  |  |  | VIII |
| 36295 | 46146 |  |  |  |  |  |  |  |  | VIII |
| 36296 | 44569 |  | 8 |  |  | 8 |  | 0.064 |  | VIII |
| 36297 | 52492 |  |  |  |  |  |  |  |  | VIII |
| 36298 | T101 |  |  |  |  |  |  |  |  | VIII |
| 36299 | 46445 |  |  |  |  |  |  |  | 1893 | II |
| 39081 | 64204 |  |  |  |  | 16 |  | 0.125 | 11366 | I |
| 39082 | 64500 |  | 0.38 |  |  | 16 |  | 0.125 | 12240 | VIII |
| 39083 | 64579 |  |  |  |  |  |  |  | 11366 | I |
| 39084 | 64605 |  |  |  |  |  |  |  | 1599 | VIII |
| 39085 | 64680 |  |  |  |  |  |  |  | 1893 | II |
| 39086 | 64586 |  |  |  |  |  |  |  | 11366 | I |
| 39087 | 64361 |  |  |  |  |  |  |  | 12241 | VIII |
| 39088 | 64755 |  |  |  |  |  |  |  | 11366 | I |
| 39089 | 64203 |  |  |  |  |  |  |  |  | I |
| 39090 | 65080 |  |  |  |  |  |  |  | 1903 | VIII |
| 39091 | 65318 |  |  |  |  |  |  |  | 1599 | VIII |
| 39092 | 65320 |  |  |  |  |  |  |  | 11366 | I |
| 39093 | 65551 |  |  |  |  |  |  |  | 12241 | VIII |
| 39094 | 65600 |  | 0.094 |  |  | 0.25 |  | 0.016 | 11750 | V |
| 39095 | 65737 |  |  |  |  |  |  |  | 11366 | I |
| 39096 | 56474 |  |  |  |  |  |  |  | 1903 | VIII |
| 39097 | 56327 |  |  |  |  |  |  |  | 1588 | VII |
| 39098 | 56682 |  | 0.094 |  |  | 3 |  |  | 1931 | V |
| 39099 | 56835 |  |  |  |  |  |  |  | 1893 | II |
| 39100 | 57568 |  |  |  |  |  |  |  | 1903 | VIII |
| 39101 | 63971 |  |  |  |  |  |  |  | 12240 | VIII |
| 39103 | 45858 |  |  |  |  |  |  |  | 1893 | II |
| 39104 | 45722 |  |  |  |  |  |  |  | 1599 | IV |
| 39105 | 44941 |  |  |  |  | 4 |  | 0.047 |  | IV |
| 39106 | 45388 |  |  |  |  |  |  |  | 12242 | I |
| 39107 | 45715 |  |  |  |  |  |  |  | 1893 | II |
| 39108 | 45910 |  |  |  |  |  |  |  |  | II |
| 39109 | 43208 -1 |  |  |  |  |  |  |  | 1903 | VIII |
| 39112 | 44271 |  |  |  |  |  |  |  |  | VIII |
| 39113 | 42876 |  | 192 |  |  | 12 |  |  | 1893 | II |
| 39114 | 44254 |  |  |  |  |  |  |  | 1903 | VIII |
| 39115 | 43306 |  |  |  |  |  |  |  | 12241 | VIII |
| 39116 | 46745 |  |  |  |  |  |  |  | 1893 | II |
| 39117 | 47244 |  |  |  |  |  |  |  |  | II |
| 39118 | 47954 |  |  |  |  |  |  |  | 1599 | IV |
| 39119 | 47547 |  | 16 |  |  | 16 |  |  | 1583 | III |
| 39120 | 47548 |  |  |  |  |  |  |  | 1893 | II |
| 39121 | 48183 |  |  |  |  |  |  |  | 12243 | I |
| 39122 | 48763 |  |  |  |  |  |  |  | 1903 | VIII |
| 39123 | 48437 |  |  |  |  |  |  |  | 1931 | V |
| 39124 | 48563 |  |  |  |  |  |  |  | 1599 | IV |
| 39125 | 48848 |  |  |  |  |  |  |  |  | IV |
| 39126 | 48213 |  |  |  |  |  |  |  | 1893 | II |
| 39127 | 48502 |  |  |  |  |  |  |  | 1599 | IV |
| 39128 | 48406 |  |  |  |  |  |  |  |  | V |
| 39129 | 50040 |  |  |  |  |  |  |  |  | V |
| 39130 | 49506 |  |  |  |  |  |  |  | 1893 | II |
| 39131 | 49676 |  |  |  |  |  |  |  | 1599 | V |
| 39132 | 49719 |  |  |  |  |  |  |  | 1893 | II |
| 39133 | 50869 |  |  |  |  |  |  |  | 8120 | V |
| 39134 | 51751 |  |  |  |  |  |  |  | 1893 | II |
| 39135 | 52057 |  |  |  |  |  |  |  | 11370 | II |
| 39137 | 65907 |  |  |  |  |  |  |  | 1599 | V |
| 39138 | 66098 |  | 0.19 |  |  | 12 |  | 0.125 | 11366 | I |
| 39139 | 42978 |  |  |  |  |  |  |  | 1893 | II |
| 40839 | Ng626 | Italy | 1 |  | 32 |  |  |  | 1901 | VIII |
| 41316 | Ng630 |  | 0.25 |  | 32 |  |  |  |  | VIII |
| 41345 | Ng632 |  | 0.23 |  | 0.008 |  |  |  | 8114 | V |
| 41346 | Ng741 |  | 0.5 |  | 8 |  |  |  |  | III |
| 41347 | Ng755 |  | 0.25 | 0.016 | 32 |  |  |  | 1582 | VIII |
| 41348 | Ng807 |  | 32 |  | 0.006 |  |  |  | 1902 | VIII |
| 41349 | Ng812 |  | 32 |  | 0.002 |  |  |  | 1584 | V |
| 41350 | Ng849 |  | 0.5 | 0.012 | 32 |  |  |  | 1582 | VIII |
| 41351 | Ng859 |  | 32 |  | 8 |  |  |  |  | VIII |
| 41352 | Ng885 |  | 0.125 | 0.006 | 3 |  |  |  |  | VIII |
| 45011 | EXNG202 | Australia |  |  |  |  |  |  | 12045 | III |
| 45012 | EXNG204 |  | 0.25 | 0.008 | <=0.03 |  |  |  | 7363 | III |
| 45015 | EXNG206 |  | 0.25 | 0.008 | <=0.03 |  |  |  | 7363 | III |
| 45016 | EXNG209 |  | 0.125 | 0.008 | <=0.03 |  |  |  | 12042 | I |
| 45017 | EXNG210 |  | 0.06 | 0.008 | <=0.03 |  |  |  | 12040 | III |
| 45018 | EXNG213 |  | 0.125 | 0.016 | <=0.03 |  |  |  |  | III |
| 45019 | EXNG219 |  | 0.125 | 0.008 | <=0.03 |  |  |  | 7363 | III |
| 45020 | EXNG214 |  | 0.125 | 0.008 | <=0.03 |  |  |  | 12045 | III |
| 45021 | EXNG225 |  | 0.125 | 0.008 | <=0.03 |  |  |  |  | III |
| 45022 | EXNG226 |  | >2 | 0.008 | 1-2 |  |  |  | 8145 | III |
| 47026 | GNCWGS_03GN_87 | China |  |  |  |  |  |  | 12039 | III |
| 47027 | GNCWGS_03SQ_89 |  |  |  |  |  |  |  | 7827 | III |
| 47028 | GNCWGS_04AV_95 |  |  |  |  |  |  |  |  | III |
| 47029 | GNCWGS_04CD |  |  |  |  |  |  |  |  | III |
| 47030 | GNCWGS_05OP |  |  |  |  |  |  |  | 11969 | III |
| 47031 | GNCWGS_061Z |  |  |  |  |  |  |  | 1583 | III |
| 47032 | GNCWGS_0712_63 |  |  |  |  |  |  |  | 1588 | VII |
| 47033 | GNCWGS_0747_64 |  |  |  |  |  |  |  | 7360 | VIII |
| 47034 | GNCWGS_075N_65 |  |  |  |  |  |  |  |  | VIII |
| 47035 | GNCWGS_07AD_68 |  |  |  |  |  |  |  | 7365 | III |
| 47347 | SRR3343472 | UK |  |  |  |  |  |  | 1580 | III |
| 47348 | SRR3343473 |  |  |  |  |  |  |  | 8122 | IV |
| 47349 | SRR3343474 |  |  |  |  |  |  |  | 1584 | V |
| 47350 | SRR3343475 |  |  |  |  |  |  |  | 1596 | IV |
| 47351 | SRR3343476 |  |  |  |  |  |  |  | 9363 | III |
| 47352 | SRR3343477 |  |  |  |  |  |  |  | 1901 | III |
| 47354 | SRR3343480 |  |  |  |  |  |  |  | 1588 | VII |
| 47355 | SRR3343481 |  |  |  |  |  |  |  | 9363 | III |
| 47356 | SRR3343482 |  |  |  |  |  |  |  | 11516 | IV |
| 47357 | SRR3343483 |  |  |  |  |  |  |  | 1584 | V |
| 54271 | LRRBGS_0039 | Brazil | 4 | 0.032 | 16 | 4 | 0.125 | 2 | 1901 | VIII |
| 54272 | LRRBGS_0040 |  | 8 | 0.032 | 16 | 4 | 0.125 | 2 |  | III |
| 54273 | LRRBGS_0041 |  | 2 | 0.015 | 4 | 1 | 0.125 | 2 |  | III |
| 54274 | LRRBGS_0042 |  | 16 | 0.015 | 4 | 32 | 0.125 | 0.5 | 1588 | VII |
| 54275 | LRRBGS_0043 |  | 4 | 0.032 | 16 | 4 | 0.125 | 2 | 1901 | VIII |
| 54276 | LRRBGS_0044 |  | 4 | 0.032 | 16 | 4 | 0.125 | 2 |  | VIII |
| 54277 | LRRBGS_0045 |  | 8 | 0.032 | 16 | 4 | 0.125 | 8 |  | III |
| 54278 | LRRBGS_0046 |  | 16 | 0.015 | 1 | 32 | 0.125 | 1 | 1588 | VII |
| 54279 | LRRBGS_0047 |  | 4 | 0.032 | 16 | 4 | 0.125 | 2 | 1901 | III |
| 54280 | LRRBGS_0048 |  | 8 | 0.064 | 16 | 4 | 0.125 | 4 |  | III |
| 59511 | KNY_NGAMR/9 | Kenya |  |  |  |  |  |  | 13614 | III |
| 60436 | KNY_NGAMR/1 |  |  |  |  |  |  |  | 13613 | VI |
| 60437 | KNY_NGAMR9p |  |  |  |  |  |  |  | 14300 | III |
| 60501 | KNY_NGAMR2 |  |  |  |  |  |  |  | 1928 | IV |
| 60502 | KNY_NGAMR3 |  |  |  |  |  |  |  | 1893 | II |
| 60503 | KNY_NGAMR4 |  |  |  |  |  |  |  | 1588 | VII |
| 60504 | KNY_NGAMR5 |  |  |  |  |  |  |  | 1599 | VIII |
| 60505 | KNY_NGAMR6 |  |  |  |  |  |  |  | 11367 | III |
| 60506 | KNY_NGAMR7 |  |  |  |  |  |  |  | 11366 | I |
| 60507 | KNY_NGAMR8 |  |  |  |  |  |  |  | 11365 | VII |
| 60508 | KNY_NGAMR10 |  |  |  |  |  |  |  | 1932 | IV |
| 60509 | KNY_NGAMR11 |  |  |  |  |  |  |  | 11365 | VII |
| 60510 | KNY_NGAMR23 |  |  |  |  |  |  |  | 13780 | VII |
| 60511 | KNY_NGAMR14 |  |  |  |  |  |  |  | 11242 | IV |
| 60512 | KNY_NGAMR20 |  |  |  |  |  |  |  | 13780 | VII |
| 60513 | KNY_NGAMR17 |  |  |  |  |  |  |  | 1921 | I |
| 60514 | KNY_NGAMR18 |  |  |  |  |  |  |  | 8133 | VII |
| 60515 | KNY_NGAMR19 |  |  |  |  |  |  |  | 1893 | II |
| 60516 | KNY_NGAMR15 |  |  |  |  |  |  |  | 13782 | VII |
| 60517 | KNY_NGAMR21 |  |  |  |  |  |  |  | 11976 | VII |
| 60518 | KNY_NGAMR22 |  |  |  |  |  |  |  | 8133 | VII |
| 60519 | KNY_NGAMR13 |  |  |  |  |  |  |  | 13782 | VII |
| 61332 | WHO_V | Sweden | >32 | 0.047 | >32 | 3 | 0.016 | >256 | 10314 | III |
| 61379 | WHO_Z | Australia | 2 | 0.5 | >32 | 3 | 1.5 | 1 | 7363 | III |
| 62968 | WHOF | Unknown |  |  |  |  |  |  | 10934 | IV |
| 88855 | WHO G | Thailand |  |  |  |  |  |  | 1903 | III |
| 88856 | WHO K | Japan |  |  |  |  |  |  | 7363 | III |
| 88857 | WHO L | Unknown |  |  |  |  |  |  | 1590 | VI |
| 88858 | WHO M | Philippines |  |  |  |  |  |  | 7367 | III |
| 88859 | WHO N | Australia |  |  |  |  |  |  | 1583 | III |
| 88860 | WHO O | Canada |  |  |  |  |  |  | 1902 | VIII |
| 88861 | WHO P | USA |  |  |  |  |  |  | 8127 | III |
| 88862 | WHO U | Sweden |  |  |  |  |  |  | 7367 | III |
| 88863 | WHO V | Sweden |  |  |  |  |  |  | 10314 | III |
| 88864 | WHO W | China |  |  |  |  |  |  | 7363 | III |
| 88865 | WHO X (H041) | Japan |  |  |  |  |  |  | 7363 | III |
| 88866 | WHO Y (F89) | France |  |  |  |  |  |  | 1901 | III |
| 88867 | WHO Z (A8806) | Australia |  |  |  |  |  |  | 7363 | III |

**References**

1. Koren, S. *et al.* Canu: scalable and accurate long-read assembly via adaptive k-mer weighting and repeat separation. *Genome Res.*  (2017). doi:10.1101/gr.215087.116

2. Li, H. Minimap and miniasm: fast mapping and de novo assembly for noisy long sequences. *Bioinformatics* **32**, 2103–2110 (2016).

3. Li, H. & Durbin, R. Fast and accurate long-read alignment with Burrows-Wheeler transform. *Bioinformatics* **26**, 589–595 (2010).

4. Li, H. *et al.* The Sequence Alignment/Map format and SAMtools. *Bioinformatics* **25**, 2078–2079 (2009).

5. Gurevich, A., Saveliev, V., Vyahhi, N. & Tesler, G. QUAST: quality assessment tool for genome assemblies. *Bioinformatics* **29**, 1072–1075 (2013).

6. Bennett, J. S. *et al.* Species status of Neisseria gonorrhoeae: evolutionary and epidemiological inferences from multilocus sequence typing. *BMC Biol.* **5**, 35 (2007).

7. Jolley, K. A., Bray, J. E. & Maiden, M. C. J. Open-access bacterial population genomics: BIGSdb software, the PubMLST.org website and their applications. *Wellcome open Res.* **3**, 124 (2018).

8. Martin, I. M. C., Ison, C. A., Aanensen, D. M., Fenton, K. A. & Spratt, B. G. Rapid sequence-based identification of gonococcal transmission clusters in a large metropolitan area. *J. Infect. Dis.* **189**, 1497–1505 (2004).

9. Demczuk, W. *et al.* Neisseria gonorrhoeae Sequence Typing for Antimicrobial Resistance, a Novel Antimicrobial Resistance Multilocus Typing Scheme for Tracking Global Dissemination of N. gonorrhoeae Strains. *J. Clin. Microbiol.* **55**, 1454 LP – 1468 (2017).

10. Unemo, M. & Shafer, W. M. Antibiotic resistance in Neisseria gonorrhoeae: origin, evolution, and lessons learned for the future. *Ann. N. Y. Acad. Sci.* **1230**, E19-28 (2011).

11. Nakayama, S.-I. *et al.* Molecular analyses of TEM genes and their corresponding penicillinase-producing Neisseria gonorrhoeae isolates in Bangkok, Thailand. *Antimicrob. Agents Chemother.* **56**, 916–920 (2012).

12. Suay-García, B. & Pérez-Gracia, M. T. Drug-resistant Neisseria gonorrhoeae: latest developments. *Eur. J. Clin. Microbiol. Infect. Dis.* **36**, 1065—1071 (2017).

13. Roberts, M. C. *et al.* Erythromycin-resistant Neisseria gonorrhoeae and oral commensal Neisseria spp. carry known rRNA methylase genes. *Antimicrob. Agents Chemother.* **43**, 1367–1372 (1999).

14. Garneau-Tsodikova, S. & Labby, K. J. Mechanisms of Resistance to Aminoglycoside Antibiotics: Overview and Perspectives. *Medchemcomm* **7**, 11–27 (2016).

15. Palace, S. G. *et al.* RNA polymerase mutations cause cephalosporin resistance in clinical Neisseria gonorrhoeae isolates. *Elife* **9**, e51407 (2020).

16. Taha, M.-K. *et al.* Multicenter Study for Defining the Breakpoint for Rifampin Resistance in &lt;em&gt;Neisseria meningitidis&lt;/em&gt; by &lt;em&gt;rpoB&lt;/em&gt; Sequencing. *Antimicrob. Agents Chemother.* **54**, 3651 LP – 3658 (2010).

17. Griffith, E. C. *et al.* The Structural and Functional Basis for Recurring Sulfa Drug Resistance Mutations in Staphylococcus aureus Dihydropteroate Synthase. *Front. Microbiol.* **9**, 1369 (2018).

18. Kivata, M. W. *et al.* gyrA and parC mutations in fluoroquinolone-resistant Neisseria gonorrhoeae isolates from Kenya. *BMC Microbiol.* **19**, 76 (2019).

19. Harrison, O. B. *et al.* Genomic analyses of Neisseria gonorrhoeae reveal an association of the gonococcal genetic island with antimicrobial resistance. *J. Infect.* **73**, 578–587 (2016).

20. Zhou, W. *et al.* Detection of gyrA and parC mutations associated with ciprofloxacin resistance in Neisseria gonorrhoeae by use of oligonucleotide biochip technology. *J. Clin. Microbiol.* **42**, 5819–5824 (2004).

21. Fiebelkorn, K. R., Crawford, S. A. & Jorgensen, J. H. Mutations in folP associated with elevated sulfonamide MICs for Neisseria meningitidis clinical isolates from five continents. *Antimicrob. Agents Chemother.* **49**, 536–540 (2005).

22. Warner, D. M., Shafer, W. M. & Jerse, A. E. Clinically relevant mutations that cause derepression of the Neisseria gonorrhoeae MtrC-MtrD-MtrE Efflux pump system confer different levels of antimicrobial resistance and in vivo fitness. *Mol. Microbiol.* **70**, 462–478 (2008).

23. Skoczynska, A., Ruckly, C., Hong, E. & Taha, M.-K. Molecular characterization of resistance to rifampicin in clinical isolates of Neisseria meningitidis. *Clin. Microbiol. Infect.* **15**, 1178–1181 (2009).

24. Lodi, L. *et al.* Neisseria meningitidis with H552Y substitution on rpoB gene shows attenuated behavior in vivo: report of a rifampicin-resistant case following chemoprophylaxis. *J. Chemother.* **32**, 98–102 (2020).

25. Chaudhry, U., Ray, K., Bala, M. & Saluja, D. Mutation patterns in gyrA and parC genes of ciprofloxacin resistant isolates of Neisseria gonorrhoeae from India. *Sex. Transm. Infect.* **78**, 440–444 (2002).

26. Cehovin, A. *et al.* Identification of Novel Neisseria gonorrhoeae Lineages Harboring Resistance Plasmids in Coastal Kenya. *J. Infect. Dis.* **218**, 801–808 (2018).

27. Lee, S.-G. *et al.* Various penA mutations together with mtrR, porB and ponA mutations in Neisseria gonorrhoeae isolates with reduced susceptibility to cefixime or ceftriaxone. *J. Antimicrob. Chemother.* **65**, 669–675 (2010).

28. Calado, J. *et al.* Antimicrobial resistance and molecular characteristics of Neisseria gonorrhoeae isolates from men who have sex with men. *Int. J. Infect. Dis.* **79**, 116–122 (2019).

29. Rouquette-Loughlin, C. E. *et al.* Mechanistic basis for decreased antimicrobial susceptibility in a clinical isolate of &lt;em&gt;Neisseria gonorrhoeae&lt;/em&gt; possessing a mosaic-like &lt;em&gt;mtr&lt;/em&gt; efflux pump locus. *bioRxiv* 448712 (2018). doi:10.1101/448712

30. Balloux, F. *et al.* From Theory to Practice: Translating Whole-Genome Sequencing (WGS) into the Clinic. *Trends Microbiol.* **26**, 1035–1048 (2018).

31. Hunt, M. *et al.* ARIBA: rapid antimicrobial resistance genotyping directly from sequencing reads. *Microb. genomics* **3**, e000131–e000131 (2017).

32. Alcock, B. P. *et al.* CARD 2020: antibiotic resistome surveillance with the comprehensive antibiotic resistance database. *Nucleic Acids Res.* **48**, D517–D525 (2019).

33. Carter, A. P. *et al.* Functional insights from the structure of the 30S ribosomal subunit and its interactions with antibiotics. *Nature* **407**, 340–348 (2000).

34. Hu, M., Nandi, S., Davies, C. & Nicholas, R. A. High-level chromosomally mediated tetracycline resistance in Neisseria gonorrhoeae results from a point mutation in the rpsJ gene encoding ribosomal protein S10 in combination with the mtrR and penB resistance determinants. *Antimicrob. Agents Chemother.* **49**, 4327–4334 (2005).

35. Singh, A. *et al.* Mutations in penicillin-binding protein 2 from cephalosporin-resistant Neisseria gonorrhoeae hinder ceftriaxone acylation by restricting protein dynamics. *J. Biol. Chem.* **295**, 7529–7543 (2020).

36. Job, V., Carapito, R., Vernet, T., Dessen, A. & Zapun, A. Common alterations in PBP1a from resistant Streptococcus pneumoniae decrease its reactivity toward beta-lactams: structural insights. *J. Biol. Chem.* **283**, 4886–4894 (2008).

37. Zapun, A., Morlot, C. & Taha, M.-K. Resistance to β-Lactams in Neisseria ssp Due to Chromosomally Encoded Penicillin-Binding Proteins. *Antibiot. (Basel, Switzerland)* **5**, (2016).

38. Beggs, G. A. *et al.* Structural, Biochemical, and In Vivo Characterization of MtrR-Mediated Resistance to Innate Antimicrobials by the Human Pathogen Neisseria gonorrhoeae. *J. Bacteriol.* **201**, (2019).

39. Olesky, M., Zhao, S., Rosenberg, R. L. & Nicholas, R. A. Porin-Mediated Antibiotic Resistance in Neisseria gonorrhoeae: Ion, Solute, and Antibiotic Permeation through PIB Proteins with penB Mutations. *J. Bacteriol.* **188**, 2300 LP – 2308 (2006).

40. Han, S. *et al.* Distinctive attributes of β-lactam target proteins in Acinetobacter baumannii relevant to development of new antibiotics. *J. Am. Chem. Soc.* **133**, 20536–20545 (2011).

41. Contreras-Martel, C. *et al.* Crystal structure of penicillin-binding protein 1a (PBP1a) reveals a mutational hotspot implicated in beta-lactam resistance in Streptococcus pneumoniae. *J. Mol. Biol.* **355**, 684–696 (2006).

42. Ma, K. C. *et al.* Increased power from conditional bacterial genome-wide association identifies macrolide resistance mutations in Neisseria gonorrhoeae. *Nat. Commun.* **11**, 5374 (2020).
